# Gliosis-dependent expression of complement factor H truncated variants attenuates retinal neurodegeneration following ischemic injury

**DOI:** 10.1186/s12974-024-03045-3

**Published:** 2024-02-22

**Authors:** Josef Biber, Yassin Jabri, Sarah Glänzer, Aaron Dort, Patricia Hoffelner, Christoph Q. Schmidt, Oliver Bludau, Diana Pauly, Antje Grosche

**Affiliations:** 1 Department of Physiological Genomics, Ludwig-Maximilians-Universität München, Planegg-Martinsried, Germany; 2 Department of Ophthalmology, University Hospital Regensburg, Regensburg, Germany; 3 Experimental Ophthalmology, University of Marburg, Marburg, Germany; 4 Institute of Experimental and Clinical Pharmacology, Toxicology and Pharmacology of Natural Products, University of Ulm Medical Center, Ulm, Germany; 5 Institute of Pharmacy, Biochemical Pharmacy Group, Martin Luther University Halle-Wittenberg, Halle, Germany

**Keywords:** Complement system, Complement factor H (FH), Retinal degeneration, Gene augmentation, Ischemic injury

## Abstract

Inherited, age-related, and acute retinal diseases are often exacerbated by an aberrant or excessive activity of the complement system. Consequently, cells not directly affected by an acute event or genetic variants may degenerate, resulting in enhanced visual impairment. The therapeutic potential of supplementation of complement factor H (FH), a key regulator of the complement cascade, is therefore particularly promising in the context of retinal diseases caused by complement activation. In this study, we engineered adeno-associated viruses (AAVs) containing sequences of two truncated human FH variants. The expression of these variants was regulated by the glial fibrillary acidic protein (GFAP) promoter, which is selectively active in gliotic Müller cells. Both FH variants consisted of FH domains 19-20, which were connected to domains 1–4 and 1–7, respectively, by a polyglycine linker. These AAVs were intravitreally injected following ischemic injury of C57BL/6J mouse retinas. We observed transgene expression in gliotic Müller cells and to some extent in astrocytes. The expression correlated directly with damage severity. Interventions resulted in decreased complement activation, accelerated normalization of microglia activity and morphological improvements. Reduced levels of *C3* transcripts and C3d protein in conjunction with higher transcript levels of inhibitory regulators like *Cfi* and *Cfh*, hinted at attenuated complement activity. This study demonstrates the great potential of complement regulatory gene addition therapy. With further in vivo testing it could be applied to treat a wide range of retinal diseases where no causative therapies are available.

**Supplementary Information:**

The online version contains supplementary material available at 10.1186/s12974-024-03045-3.

## Introduction

Retinal diseases, such as age-related macular degeneration (AMD), diabetic retinopathy, glaucoma and uveitis, are leading causes of vision loss and blindness across all demographics and age groups [2, 39, 64]. Despite their different pathological mechanism, these diseases share a common feature: the involvement of the complement system in their development and/or progression [11, 25, 39, 40, 43]. However, effective treatments that address their underlying causes of these diseases are still limited. Therefore, targeting the complement system represents a promising approach for the treatment of these devastating conditions.

The complement system is an essential part of the innate immune response. It is responsible for recognizing and eliminating foreign particles such as bacteria and viruses, as well as apoptotic or modified host cells [3]. Complement components are continuously secreted into the circulation and are also locally expressed in immune-privileged tissues such as the retina [13, 35, 49, 70]. In the retina, inflammation in early disease stages is primarily driven by local players [3], including astrocytes, microglia and Müller cells. These cells contribute to the maintenance of complement homeostasis and are primary suppliers of key pro-inflammatory factors [37, 67].

**Fig. 1:**
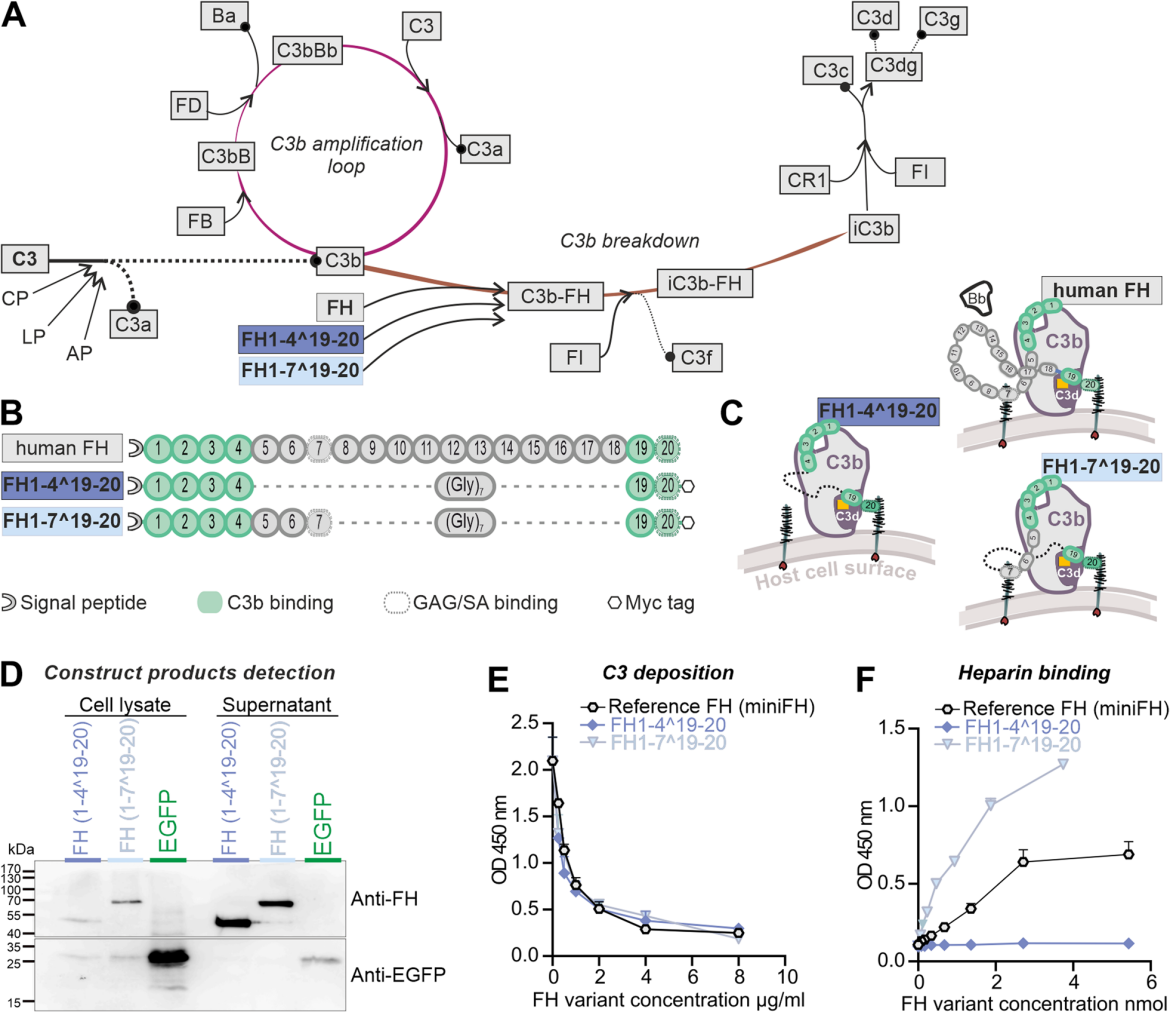
Mechanisms of FH in the complement regulation, illustration  of FH variant structures and in vitro efficacy evaluation. GAG: Glycosaminoglycans, SA: Sialic acid, FH: Factor H, Bb: C3-binding fragment of complement factor B, FI: Factor I, FD: Factor D, Cr1: Complement C3b/C4b Receptor 1, CP: Classical Pathway, AP: Alternative Pathway, LP: Lectin Pathway**. A** The complement system is activated by three pathways: the classical pathway (CP), the lectin pathway (LP), and the alternative pathway (AP). These pathways converge in an amplification loop of the AP through the formation of C3b. FH binds to C3b and prevents the interaction of FB and its activated cleavage product Bb, thereby interrupting the amplification loop. This is known as the decay acceleration function because the C3 convertase (C3bBb) is not formed. In addition, FH acts as a cofactor for the serine protease FI that facilitates the breakdown of C3b into smaller cleavage products (iC3b, C3f). This process is crucial for the complete degradation of C3b by FI and its cofactors, such as CR1. **B** FH comprises of 20 CCP domains, with CCP1-4 and CCP19-20 mediating C3b binding. Binding to cell surfaces via glycosaminoglycans (GAG) primarily occurs through the CCP7 and CCP20 domains. The truncated FH variants used, consist of either the FH domains CCP1-4 and CCP19-20, which are connected by a glycine linker (FH1-4^19-20) or the domains CCP1-7 and CCP19-20 (FH1-7^19-20). Both truncated FH variants have a c-terminal signaling peptide for secretion and an n-terminal epitope (myc) tag. **C** FH inhibits the formation of C3 convertase (C3bBb) and accelerates C3bBb decay by binding the CCP1-4 domain to C3b and displacing Bb (upper part). Both FH1-4^19-20 and FH1-7^10–20 are capable of performing this FH function (lower part). Additionally, FH anchors to the cell surface through GAG binding, with FH1-4^19-20 having one such binding site and FH1-7^19-20 having two. **D** FH1-4^19-20 and FH1-7^19-20 were expressed and secreted by HEK293 cells. Both truncated FH variants were found with their predicted size in Western blot. EGFP was predominantly detected in cell lysates, corroborating that the FH1-4^19-20 and FH1-7^19-20 proteins were secreted and not released due to cell ruptures. Original documentation of the blots is added as Additional file 1: Fig. S1. **E** Upon addition of truncated FH variants to mouse serum, reduced C3b deposition was observed on AP-activating lipopolysaccharide-coated microtiter plates. This indicates that the truncated FH variants can mediate the decay acceleration function of FH. Each data point represents the mean ± SEM for n = 2 (biological replicates, shown is one of two technical replicates with similar results). **F** The evaluation of GAG binding revealed that FH1-7^19-20 exhibited the strongest binding affinity for immobilized heparin. In comparison to FH1-7^19-20, the reference miniFH of [57] demonstrated a lower binding capacity. FH1-4^19-20 did not show any interactions, likely due to a myc tag induced blockade in the CCP20 GAG binding domain. Each data point represents the mean ± SEM for n = 2 (biological replicates, shown is one of two technical replicates with similar results)

The complement cascade is initiated when complement recognition molecules bind to triggers such as antibody-antigen complexes or foreign particle surfaces [3, 38]. This cascade involves a series of serine proteases that lead to cell lysis, the generation of anaphylatoxins and opsonization markers. The cleavage of C3 into C3a and C3b by C3 convertases is a pivotal part of this process (Fig. 1A) [3, 38]. This self-reinforcing response is negatively regulated by membrane-bound and soluble regulators, such as complement factor H (FH). FH is a soluble glycoprotein consisting of 20 complement control protein (CCP) domains that recognize self-structures in the form of glycosaminoglycans (GAG) as well as C3b [4] (Fig. 1B, C). FH regulates complement activation by acting as a cofactor for complement factor I (FI), destabilizing the C3bBb convertase, and competitively binding C3b. Cofactors are essential to enable FI to hydrolyse C3b into iC3b, and subsequently to C3dg and C3d (Fig. 1A) [10, 51, 71]. FH also undergoes conformational changes upon binding to C3b, which enhance its regulatory activity and promote its interaction with other complement regulators [47].

This complement cascade regulation by cleavage of C3b is favored on self (in contrast to foreign) surfaces [29, 41]. Structurally, the regulatory function on C3b in the fluid phase is mainly facilitated by FH CCP1-4, whereas the domains 19-20 are involved in GAG recognition and obstructing the thioester domain (TED) of C3d [28, 41, 66]. Multiple polymorphisms in the *CFH* gene have been associated with AMD [17, 68]. The pathology underlying *CFH* variants such as the Y402H polymorphism in CCP7 of *CFH*, where carriers have a 5.2-fold increased risk of developing AMD is still under investigation [30, 62]. Functionally, CCP7 and directly neighboring domains were shown to possess GAG binding properties [24, 56].

Several studies aimed at replacing or substituting FH have been conducted with promising results [5, 18, 46]. Schmidt et al. previously reported the successful engineering of a size reduced FH in which domain sets 1–4 and 19-20 are linked by a polyglycine linker, providing the flexibility to exert all regulatory functions and even enhancing the recognition of C3b degradation products by FH CCP19-20 [57]. This approach presented an upstream complement regulator that acts on the cell surface and fluid phase C3 convertase and stops the amplification of the complement response while minimizing the potential negative effects of long-term inhibition of the complement system [57].

In this study, we investigated the capabilities of two truncated versions of human FH in the murine ischemia/reperfusion model. During an ischemic event, inadequate vascular supply leads to nutrient and oxygen deprivation causing neurodegenerative damage in the retina. Ischemia and subsequent reperfusion result in inflammatory and oxidative stress, leading to microglial activation and degeneration of all retinal layers and the optic nerve [52, 53, 65]. This degenerative process is characterized by changes in the expression of cell-specific proteins, including complement components [49].

The FH constructs were successfully delivered using adeno-associated viruses (AAV) and retain key functions necessary for host cell recognition and cofactor activity. We detected transgene activity primarily in gliotic Müller cells and to a lesser extent in astrocytes, with the level of expression correlating with the severity of the damage. The treatments resulted in reduced complement activation, faster stabilization of microglia, and structural improvements. Additionally, we observed lower levels of *C3* transcripts and C3d protein, along with increased expression of inhibitory regulators, altogether indicating attenuated complement activity.

## Results

### Truncated FH variants retain full length FH functions* in vitro*

We modified the miniFH regulator, originally developed by Schmidt et al., for expression in mice [57]. This involved codon optimization and the addition of both a signal peptide and an epitope (myc) tag. MiniFH is derived from FH CCP1-4 and 19-20 domains of human FH (Fig. 1B). Replacement of the 14 middle domains with a polyglycine linker provided structural advantages but eliminated some functions, notably the GAG recognition of CCP6-7 domains. The importance of the CCP7 domain, which contains the Y402 locus, was underscored by its recent identification as critical for the antiangiogenic functions of FH in a choroidal neovascularization model [6]. Therefore, we introduced a second construct that combined the benefits of domain removal with the preservation of domains critical for tissue maintenance in AMD. Both truncated FH versions were within the loading capacity of an AAV. FH1-4^19-20 and FH1-7^19-20 were designed to exert important regulatory functions: CCP1-4 domains facilitated C3b binding and cofactor activity, whereas CCP6-7 and 19-20 domains ensured polyanion binding (Fig. 1B). The hypothesis was that these truncated FH variants mimic the regulatory functions of native FH by maintaining host cell recognition and competition for complement factor B (FB/Bb) binding sites on C3b (CCP1-4) (Fig. 1C).

**Fig. 2:**
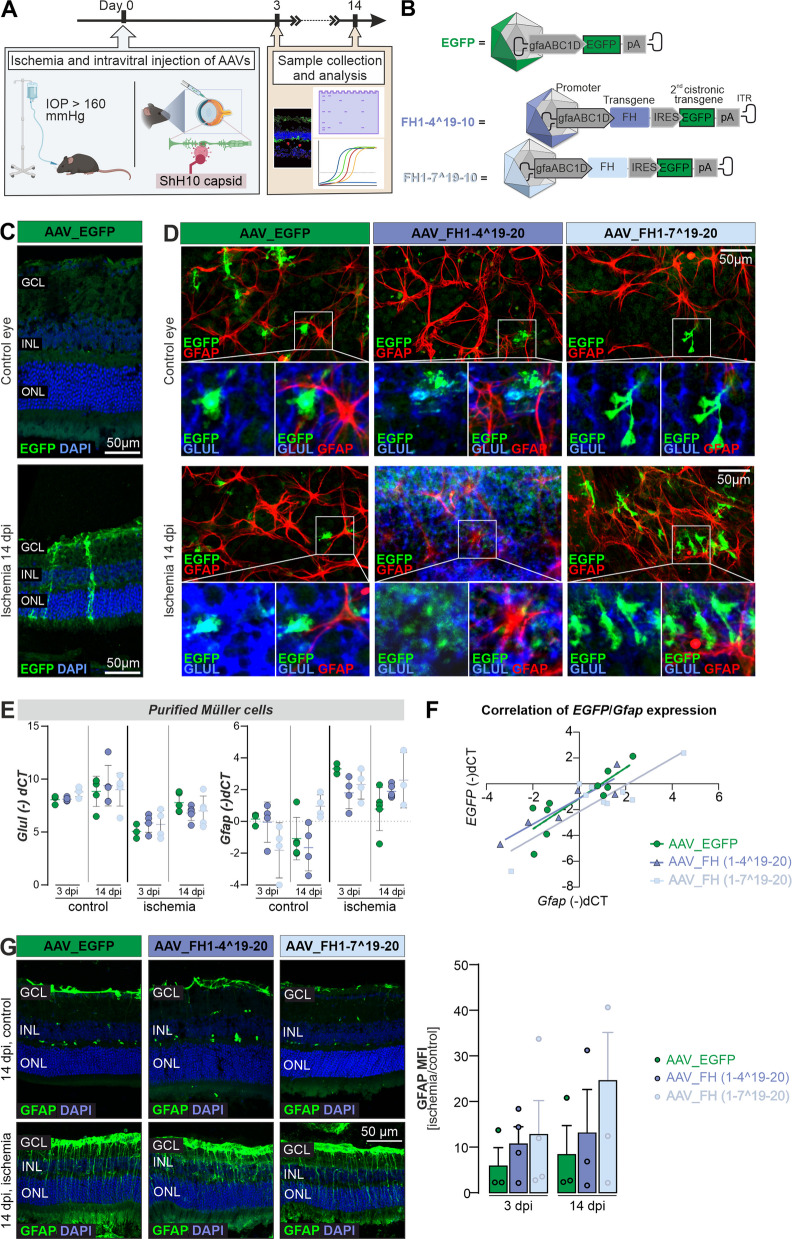
Effectiveness of the expression cassette with regard to coupling to GFAP transcription and Müller cell specificity in vivo. **A** Experimental set up for in vivo studies: eyes of C57BL/6 J wildtype mice were exposed to hypoxic conditions by elevated intraocular pressure (IOP), resulting in ischemic tissue responses. Subsequently, AAV vectors carrying expression cassettes for regulatory truncated FH variants and an EGFP-only control were injected. Tissue samples were collected for evaluation at intervals of 3 and 14 days post injections (dpi). **B** Scheme delineating the viral constructs used in the present study. **C** Cryosections of the central retina showed that EGFP was present in Müller cells identifiable by their unique morphology spanning the whole tissue from the ganglion cell layer (GCL) to the outer border of the outer nuclear layer (ONL). This indicated a cell type-specific expression of the AAV construct mainly in Müller cells. **D** Immunohistochemistry (IHC) analysis of peripheral retinal flat mounts at 14 dpi at the level of the nerve fiber layer after ischemic injury and AAV application was performed to assess whether also astrocytes, that even in the healthy retina express high levels of GFAP, were transduced by the AAV. EGFP-positive cells co-expressed the Müller cell marker glutamine synthetase (Glul), but not high levels of GFAP. In contrast, highly GFAP-positive astrocytes were not highlighted by EGFP-labeling (also see Additional file 1: Fig. S2). **E** Quantitative real-time PCR of isolated Müller cells showed a decrease in *Glul* expression in ischemic Müller cells at 3 dpi (left panel). This reduction is attenuated by 14 dpi. In contrast, *Gfap* expression increased by 3 dpi and remained elevated until 14 dpi (right panel). Each data point per biological replicate (n = 3–4) is represented by a dot in the graph. **F** *EGFP* expression levels showed correlation to *Gfap* expression in all three treatment groups as determined by qPCR (14 dpi). Pearson’s correlation coefficients of *Gfap* (-)dCT vs *EGFP* (-)dCT were as follows: *ρ*_(AAV_EGFP)_ = 0.8561, *ρ*_(AAV_FH1-4^19-20)_ = 0.8561 and *ρ*_(AAV_FH1-7^19-20)_ = 0.8534. Each data point per biological replicate (n = 3–4) is represented by a dot in the graph. **G** Representative microscopic images showing GFAP immunostaining on sections at 14 dpi (left panel). Quantitative analysis of mean fluorescence intensity encompassing the retina from the outer limiting membrane to the inner plexiform layer (right panel). The ganglion and nerve fiber layers were excluded from the analysis to eliminate signals from astrocytic GFAP expression. Each data point per biological replicate (n = 3–4) is represented by a dot in the graph

Both variants were expressed using a mammalian expression system for the in vitro studies. FH1-4^19-20 and FH1-7^19-20 proteins were detected in cell lysates and supernatant of transfected HEK293 cells by Western blot and had the predicted sizes of 44 and 68 kDa, respectively (Fig. 1D). The control reporter protein, enhanced green fluorescent protein (EGFP) without signal peptide for secretion, was found mainly in cell lysates. This observation confirmed that the truncated FH proteins were actively secreted and not released by cell disruption (Fig. 1D, Additional file 1: Fig. S1). Evaluation of protein levels of the expressed truncated FH proteins compared with EGFP revealed that bicistronic mRNAs carrying the FH proteins and the EGFP reporter signal exhibited reduced expression under internal ribosome entry site (IRES) translational control than the single cistron in the EGFP control vector (Figs. 1D, 2B).

Previously, miniFH variants were reported to effectively inhibit human C3b deposition on cell surfaces, suggesting that they modulate the complement pathway [57]. Our matched FH1-4^19-20 and FH1-7^19-20 variants mirrored the dose-dependent ability of the reference miniFH to prevent complement accumulation in mice, as evidenced by the reduction of C3b deposition by mouse serum on a lipopolysaccharide-coated surface (Fig. 1E).

CCP7, which was previously found to bind GAGs [9], led to the expectation that FH1-7^19-20 would have an enhanced ability to recognize cell surfaces through GAG binding. This assumption was confirmed indirectly by a heparin-binding ELISA (Fig. 1F), in which FH1-7^19-20 showed the highest binding strength for heparin among all FH variants. In contrast, FH1-4^19-20 did not show any detectable interactions in this context (Fig. 1F). Because the structure of this protein is identical to that of the reference miniFH, with the exception of an epitope tag at the C-terminus, this lack of interaction was likely due to an obstruction in the CCP20 GAG-binding domain.

### Expression system is Müller cell-specific and GFAP dependent

After demonstrating that the recombinant FH variants retained most of the full-length functions of FH, with the exception of GAG binding of FH1-4^19-20 (Fig. 1), we tested the influence of the constructs in vivo using the acute ischemia/reperfusion retinal mouse model [49] (Fig. 2A). Note that the goal of our study was to focus on two distinct phases of retinal ischemia/reperfusion: the acute phase of maximal inflammation, possibly involving complement activation, at 3 days post injury (dpi), and a later phase at 14 dpi, characterized by tissue remodeling and restoration of tissue homeostasis after the initial injury [1, 36, 48, 49].

To protect retinal neurons from transgene expression and focus the response on the risk of impending retinal degeneration, we targeted the expression of the secreted factors to Müller cells. This was achieved by generating AAVs with the ShH10-capsid with reported tropism for Müller cells [31]. In addition, we aimed to link peak transgene expression to Müller cell gliosis by including an optimized version of the human glial fibrillary acidic protein (GFAP) promoter into the expression cassette (Fig. 2B). After ischemia induction, AAVs were injected intravitreally to access Müller cell end feed adjacent to the vitreous, which form the inner blood retinal barrier (Fig. 2A). It should be noted that astrocytes are also located in the nerve fiber layer and could potentially be easily transduced by AAVs injected intravitreally as well. Analysis of the pattern of EGFP expression in retinal sections (Fig. 2C) and flat mounts (Fig. 2D, Additional file 1: Fig. S2) confirmed: (i) widespread transgene expression throughout the retina from the central areas to the far periphery (Additional file 1: Fig. S2A), (ii) substantial transgene expression as early as 3 days after ischemia and AAV delivery (dpi) (Additional file 1: Fig. S2), (iii) GFAP expression in astrocytes, but also in Müller cells, even in control eyes, possibly due to the tissue stress caused by intravitreal delivery of AAV (Additional file 1: Fig. S2B), (iv) an EGFP expression pattern mainly restricted to glutamine synthetase (GLUL)-positive Müller cells, with few GFAP-positive astrocytes showing low levels of EGFP in control eyes, but a large number of transgene-positive Müller cells and many EGFP-positive astrocytes in the post-ischemic retina, and finally, (v) that the AAV constructs for both truncated FH variants resulted in detectable EGFP expression at 14 dpi (Fig. 2D).

For molecular validation of those findings, retinal samples were collected 3- and 14-days post-ischemia (dpi), and retinal cell types were separated by magnetic-activated cell sorting (MACS). Successful enrichment of each cell population was demonstrated analyzing the mRNA expression of known marker genes (Additional file 1: Fig. S3). The rise in  *Gfap* transcript levels in control eyes, matching our immunolabeling results, suggests a temporary gliotic response from intravitreal AAV injection. This is consistent with reports from others demonstrating a temporarily confined gliotic response of both Müller cells and microglia in mouse eyes to intravitreal injections [59, 60]. We analyzed the effects of AAVs carrying the truncated FH variants on ischemia/reperfusion hallmarks in purified Müller cells by qPCR. A previously reported decrease in *Glul* levels after ischemia/reperfusion was reproduced in the present study (Fig. 2E). However, we observed no detectable treatment effect of AAVs on this Müller cell marker (Fig. 2E) [44, 65].

As a marker of Müller cell gliosis, *Gfap* levels increased at 3 dpi and decreased 14 days after injection of AAV_EGFP-control vector (Fig. 2E). After injection of AAV_FH1-4^19-20 or AAV_FH1-7^19-20, there were no significant shifts in *Gfap* expression between 3 and 14 dpi. However, a slight trend suggestive of reduced *Gfap*-upregulation at 3 dpi was observed in Müller cells from ischemic retinas treated with FH variants compared to those treated with EGFP alone, an effect that diminished at 14 dpi (Fig. 2E). This could be a hint of reduced gliosis and might indicate that the treatment is effective in mitigating the cellular response to the injury.

Furthermore, analysis of *EGFP* and *Gfap* transcript levels at 14 dpi revealed a significant relationship between *Gfap* dCT and *EGFP* dCT values of AAV_EGFP, AAV_FH1-4^19-20 and AAV_FH1-7^19-20 (Fig. 2F). This suggests that expression of the transgenes is indeed coupled to that of *Gfap*. Finally, GFAP concentrations determined from immunostainings intensity scores did not differ significantly between treatments or time points (Fig. 2G).

### Positive staining of microglia in ischemic retinas after treatment with AAV_FHs

**Fig. 3:**
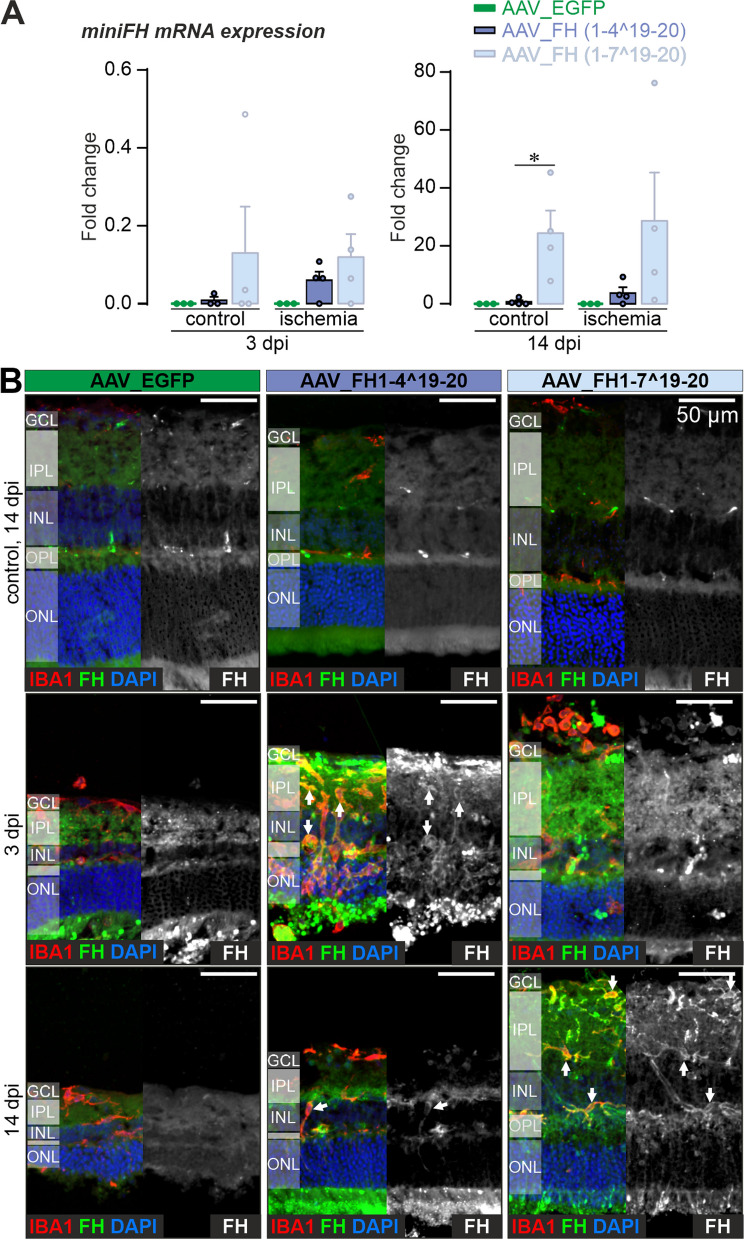
Expression of native and transgenic FH variants at transcript and protein levels. **A** *Left*, at 3 dpi, mRNA expression levels of regulatory *CFH* variants were already detectable in the Müller cell fraction, but at low levels. *Right*, after 14 days, a stronger expression of both FH1-4^19-20 and FH1-7^19-20 mRNA variants was observed in purified Müller cells. Bars represent the mean ± SEM of n = 3–4 biological replicates. Each data point is represented by a dot in the graph. Unpaired t-test: *P < 0.05. **B** IHC results from cryosections of control and post-ischemic retinas after AAV treatment delineating FH deposition and the localization of microglia/macrophages by a labeling for IBA1 at 3 and 14 dpi. FH, factor H. Scale bar, 50 µm

In the early phase of the observation period, specifically at 3 dpi, FH variant transcripts were detectable only in trace amounts in the Müller cell fraction of both ischemic and control eyes (Fig. 3A). However, after 14 dpi, mRNA levels of FH variants were increased in Müller cells, indicating not only robust expression but also interanimal variability (Fig. 3A).

Following the ischemic injury, there was pronounced activation of microglia that spread through the whole retina (Fig. 3B). Interestingly, immunoreactivity with an antibody against FH was observed in retinas treated with AAV_FH1-4^19-20 and AAV_FH1-7^19-20 (Fig. 3B). It is important to note that although this antibody can also identify mouse FH, no microglia-specific staining was seen in eyes injected with the AAV_EGFP control. The microglia-specific FH signal was also evident at 14 dpi. Of note, blood vessels were also stained, likely due to nonspecific binding of the secondary antibody to endogenous mouse IgG, a phenomenon also observed in control staining with secondary antibody (Additional file 1: Fig. S4).

### FH variants attenuate tissue damage in retinal ischemia/reperfusion model

**Fig. 4:**
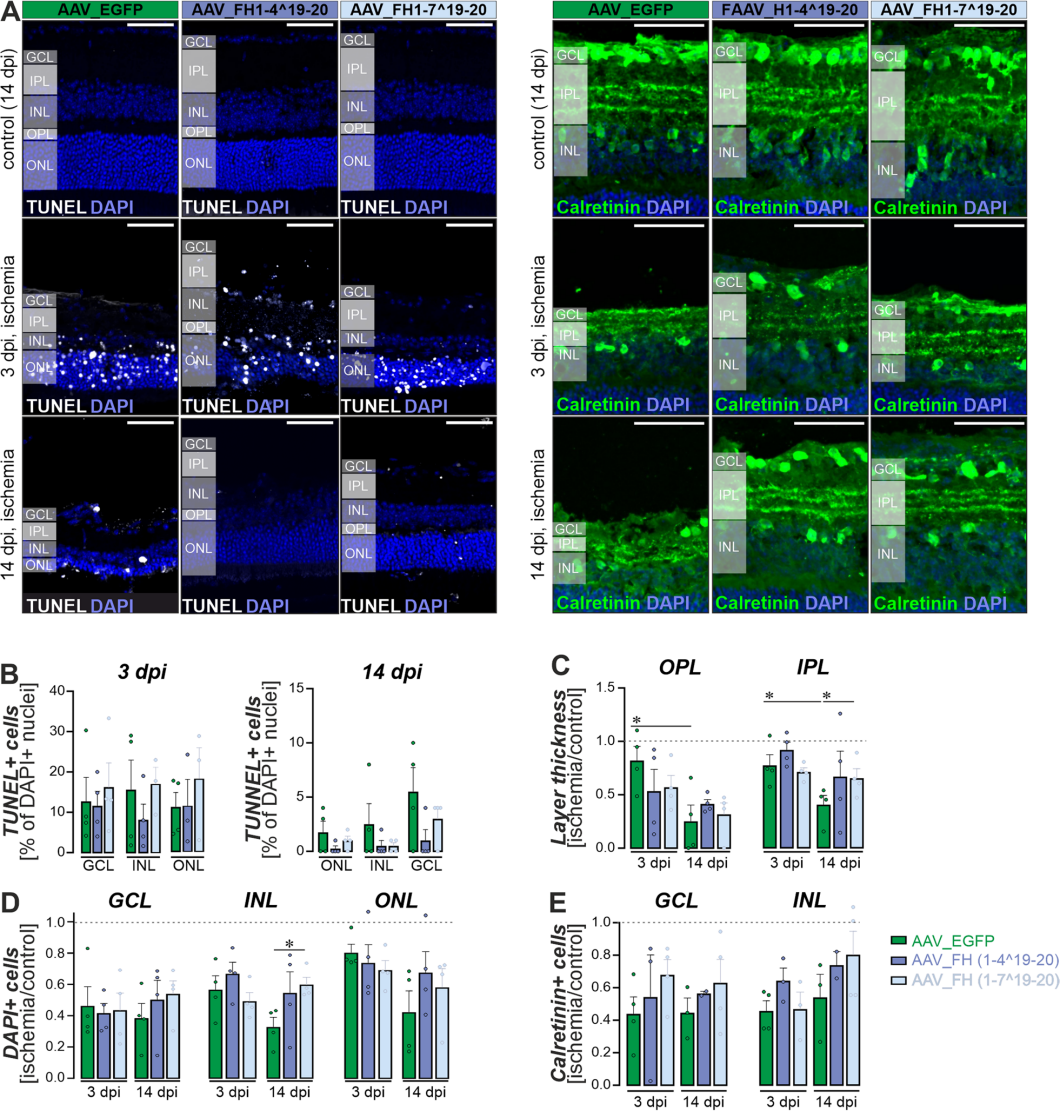
Characterization of neuronal survival in the ischemia/reperfusion model following treatment with AAV_EGFP, AAV_FH1-4^19-20 and AAV_FH1-7^19-20 (n = 3–6). **A** Cryosections from the central retina revealed TUNEL-positive apoptotic/necrotic cells (left panel) and calretinine-positive amacrine and ganglion cells (right panel) in both non-ischemic control and ischemic eyes treated with AAVs. **B** Quantitative analysis of the percentage of TUNEL-positive cells in each retinal layer and treatment at 3 and 14 dpi. **C** Evaluation of the outer plexiform layer (OPL) and the inner plexiform layer (IPL) thickness to assess the integrity of the synaptic connections representing neuronal processes. Values were normalized to non-ischemic control eyes of the corresponding animals. **D**. Quantification of nuclei within the ganglion cell layer (GCL), the inner nuclear layer (INL) and the outer nuclear layer (ONL) per scan field. Data were normalized to non-ischemic control eyes of the corresponding animals. **E** To examine cell-specific neuronal loss in more detail, particularly given the susceptibility of inner retinal neurons to ischemia/reperfusion, we quantified calretinin-positive cells. Calretinin labels ganglion cells as well as displaced amacrine cells in the GCL and amacrine cells in the INL. **B–E** Each data point per biological replicate (n = 3–4) is represented by a dot in the graph. Unpaired t-test: *P < 0.05. **C–E** Dashed lines indicate the level of each parameter in the non-ischemic control eye.

Three days after ischemic injury, TUNEL staining revealed apoptosis and/or necrosis in approximately 13% of cells in the ganglion cell layer (GCL), 16% of cells in the inner nuclear layer (INL), and 11% in the outer nuclear layer (ONL) of AAV_EGFP injected eyes (Fig. 4A, B). At 14 dpi, these numbers decreased to less than 5% for the GCL and less than 1% of cells were TUNEL-positive in the INL or ONL. Despite a trend toward overall lower numbers of TUNEL-positive cells, the AAV_FH1-4^19-20 and AAV_FH1-7^19-20 treatments did not show a statistically significant effect in this analysis (Fig. 4B).

Consistent with the TUNEL data, the ischemic episode resulted in severe disruption of the retinal architecture and substantial loss of cell nuclei in all nuclear layers in eyes receiving the AAV_EGFP control construct: approximately 54% in the GCL, 44% in the INL and 19% in the ONL, respectively (Fig. 4A, D). This degeneration appeared to progress after 3 dpi, as evidenced by even greater losses at 14 dpi especially in the ONL (Fig. 4D). In line with this, also the outer (OPL) and inner (IPL) plexiform layer thickness was significantly reduced at 14 dpi (Fig. 4C).

Although all morphometric parameters tested are closer to 1 (i.e., equal to the values in the non-ischemic control eye), the therapeutic benefits of AAV_FH1-4^19-20 and AAV_FH1-7^19-20 did not reach statistical significance compared to ischemic eyes treated with AAV_EGFP (Fig. 4C, D). It is important to note, however, that there was no significant progression of structural damage in the post-ischemic retinas treated with either of the FH variants, as was observed in the plexiform layers of the AAV_EGFP-treated controls (Fig. 4C). Consistent with these findings, the AAV_FH1-7^19-20 injected retinas not only exhibited significant improvement in neuronal survival in the INL, as evidenced by a 1.8-fold increase in nuclei count compared to the AAV_EGFP group at 14 dpi (Fig. 4D), but also demonstrated enhanced IPL thickness (Fig. 4C). Animals treated with AAV_FH1-4^19-20 also showed similar positive trends, though these were not statistically significant.

Previous studies have shown that amacrine and ganglion cells are particularly vulnerable to retinal ischemia [32]. In our study, we found a clear stratification into three distinct layers of calretinin-positive dendrites in the IPL, with calretinin-positive amacrine cell bodies being located in the INL and those of ganglion cells in the GCL (Fig. 4A, E). After 3 dpi, ischemia resulted in a loss of approximately 60% of ganglion and amacrine cells. The calretinin-positive cells of the AAV_FH-treated retinas showed higher, but not yet significantly improved, cell survival rates. However, it was observed that the treatments with both FH variants contributed to enhanced structural integrity of calretinin-positive dendrites in the IPL, maintaining the three characteristic layers (Fig. 4A), aligning with our earlier findings about the plexiform layer’s sensitivity to our treatment approach (Fig. 4C).

In summary, the morphometric data indicate that both FH variants may effectively mitigate or prevent secondary cell death. This reduction is likely attributable to the dampening of excessive inflammatory responses, which are partly driven by complement activation following the initial extensive cell loss from direct ischemic injury.

### AAV_FH treatments modulate the pre- and post-ischemic complement transcriptome

**Fig. 5:**
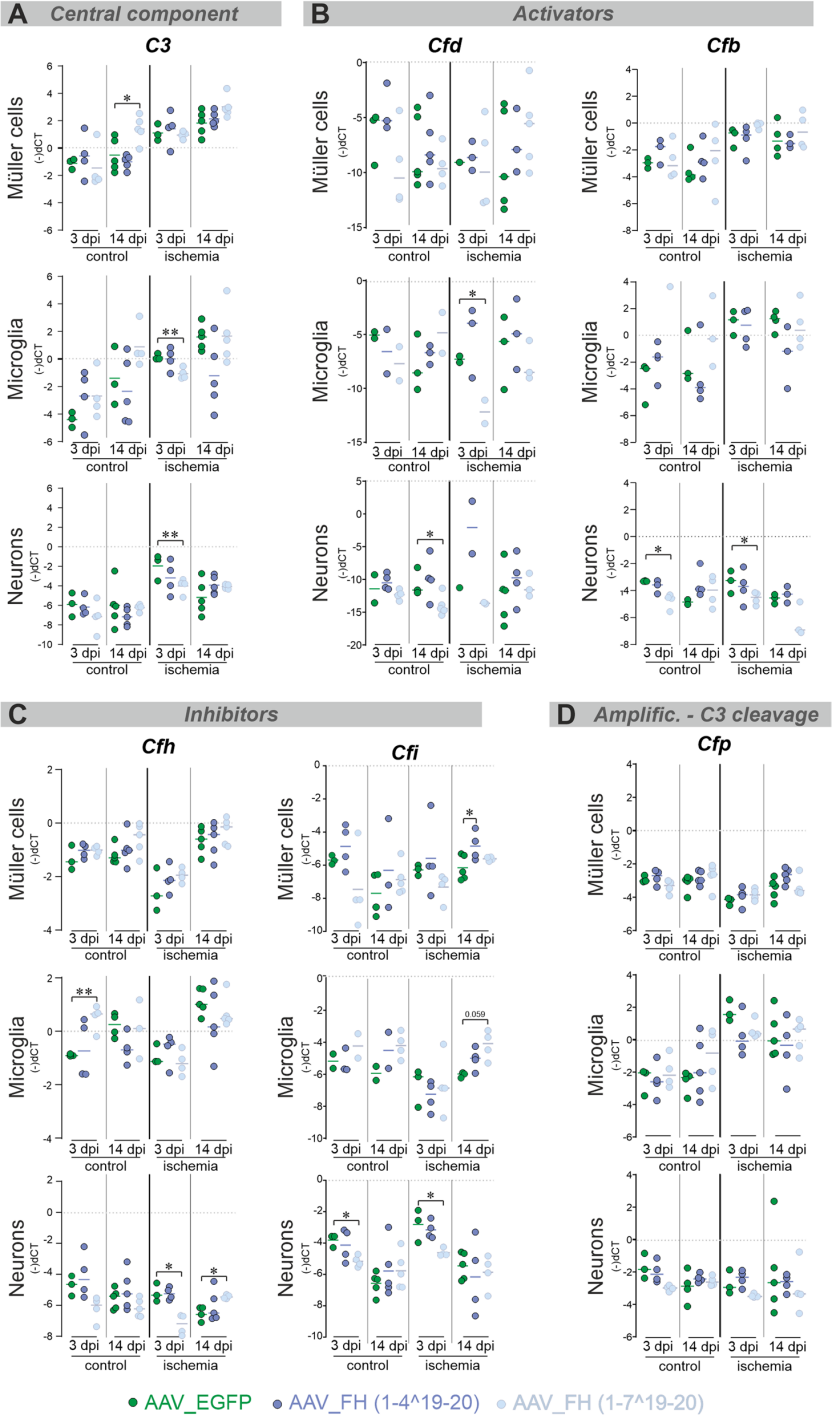
Cell type-specific analysis of complement component expression in the murine retina 3 and 14 dpi. qPCR data are presented as negative delta Ct values, i.e. that high numbers represent higher transcript levels on a logarithmic scale. Neuronal, microglia and Müller cell fractions were analyzed for transcripts of **(A)** the central component *C3*,** (B)** complement components involved in activation *Cfd* and *Cfb* or **(C)** inhibition of the complement cascade *Cfh* and *Cfi* and **(D)** *Cfp* that is involved in the amplification of C3 cleavage**.** Each data point per biological replicate (equals the number of mice studied: n = 2–5) is represented by a dot in the graph. Unpaired t-test: *P < 0.05; **P < 0.01. (All statistical analyses are available in Additional file 1: Tables S1, S2, and S3.)

Comparison of control eyes treated with AAV_EGFP, AAV_FH1-4^19-20 and AAV_FH1-7^19-20 with their ischemic counterparts revealed marked differences in complement expression. These differences reflect the distinct ischemia-associated immunological phenotype previously described [49]. Increased *C3* mRNA levels were observed in purified Müller cells of all ischemic treatment groups at 14 dpi and to some extent in the neuronal fraction at 3 dpi compared with their non-ischemic counterparts (Fig. 5A, Additional file 1: Table S3). The complement activating factors *Cfd* and *Cfb* showed increased expression in ischemic eyes in all treatment groups, although differences manifested in different cell fractions and time points (Fig. 5B, Additional file 1: Tables S1, S2). Of note, innate murine *Cfh* was consistently downregulated in Müller cells and neurons after ischemia, regardless of whether the animals received AAV_FH-treatment. In contrast, *Cfi* increased specifically in neurons at 3 dpi (Fig. 5C, Additional file 1: Table S3). Following ischemia, stabilizer of the alternative pathway C3 convertase *Cfp* was downregulated in Müller cells but upregulated in microglia as observed before (Fig. 5D, Additional file 1: Table S3) [49].

To identify the specific effects of AAV_FH gene addition therapy, we compared the expression levels of complement components in Müller cells, microglia and neurons from ischemic eyes with those receiving the AAV_EGFP control virus (Additional file 1: Tables S1, S2).

When examining the effects of the AAV_FH1-7^19-20 variant treatment, we observed notable differences in complement expression compared to the AAV_EGFP control group. Even when comparing within control eyes, these differences were apparent (Fig. 5A–C). These effects leaned towards complement downregulation, with lower transcript levels of complement activators (Fig. 5B) and higher levels of inhibitors (Fig. 5C). For instance, activators *Cfb* and *Cfd* were less expressed in neurons (Fig. 5B) and more *Cfh* transcripts were found in microglia (Fig. 5C). On the other hand, Müller cells of control eyes expressed higher levels of the central complement component *C3* after treatment with AAV_FH1-7^19-20 (Fig. 5A)*.* This was interesting, since C3 was downregulated three days after ischemia in microglia and neurons with AAV_FH1-7^19-20 treatment (Fig. 5A).

Post-ischemic neurons from retinas treated with AAV_FH1-7^19-20 showed less *Cfh* transcripts 3 dpi (Fig. 5C), which flipped to an upregulation 14 dpi. Similar to *Cfh,* the C3b protease *Cfi* was also downregulated 3 days after ischemia when treated with AAV_FH1-7^19-20. For AAV_FH1-4^19-20 treatment an upregulation of *Cfi* in Müller cells 14 days after ischemia was observed (Fig. 5C).

Transcript levels of the terminal pathway components *C9* and *C5* were also assessed. However, as demonstrated recently [49] these transcripts were mostly out of assay range and no valid comparison could be made (data not shown).

### Post-ischemic retinas treated with AAV_FH constructs showed less C3 turnover and complement activation

**Fig. 6:**
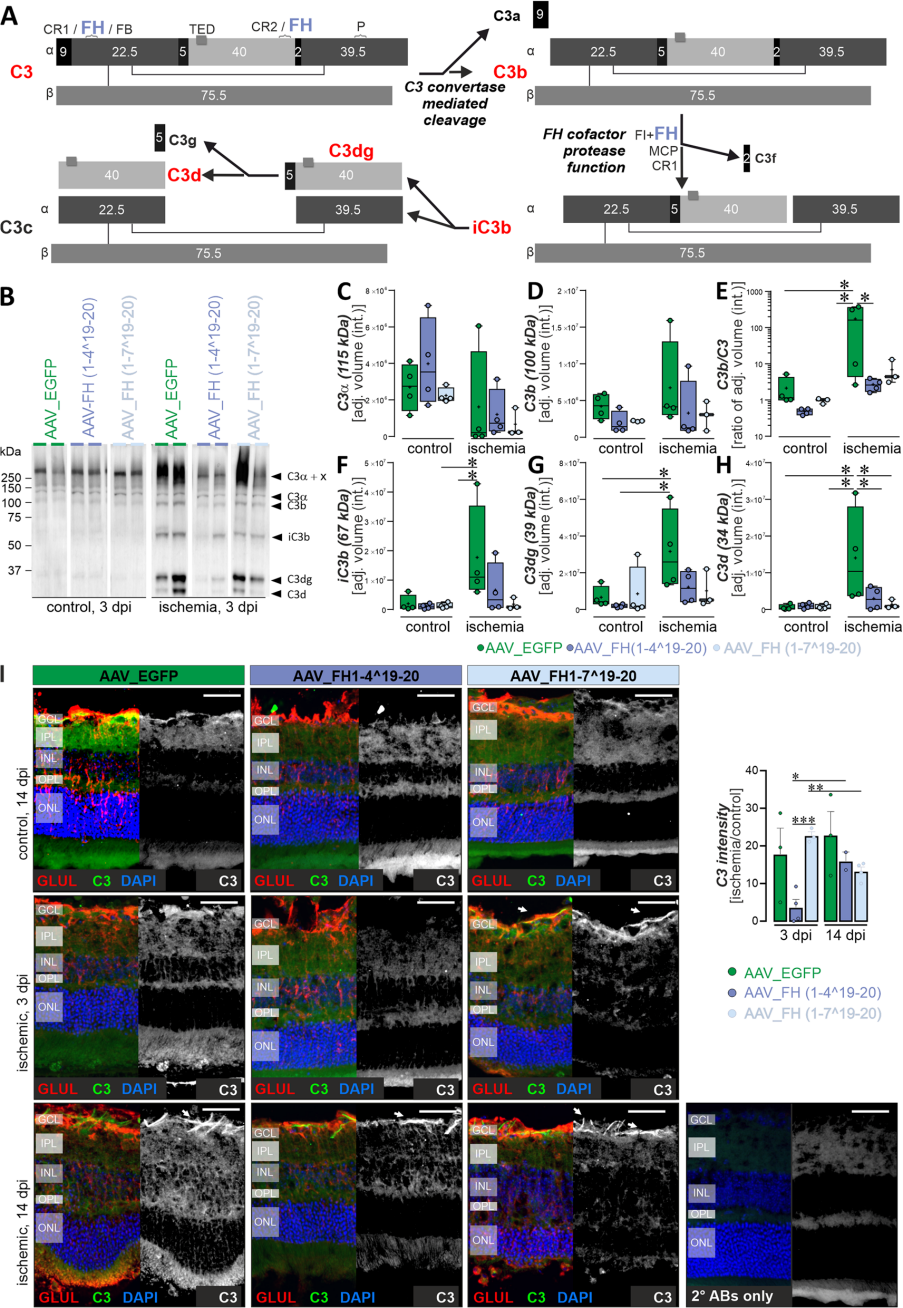
Quantitative analysis revealed that truncated FH variants modulate C3 fragmentation in the ischemic retina. **A** Schematic representation of C3 cleavage. Numerical data indicate peptide sizes in kilodalton (kDa). The binding sites for complement receptors 1 and 2 (CR1 / CR2), FH, MCP and factor B (FB) are highlighted. C3 convertase cleaves C3 into C3b and C3a. With MCP, CR1 and FH as cofactors cleaves FI C3b into iC3b and C3f. Subsequent cleavage of iC3b by FI leads to C3c and C3dg, the latter being further converted to C3d. Modeled after [34]. **B** Representative Western blot of AAV treated retinas containing 70 µg total protein. Detection was performed by using a polyclonal antibody specific for C3d. (C3α + x corresponds to C3 α-chain covalently attached to glycoproteins via thioester group.) Original documentation of the blot is shown in Additional file 1: Fig. S5. **C–H** Quantitative analysis of Western blot band intensities normalized to total protein loading. Boxplots represent median, quartiles and mean (indicated by a dot) without outliers. The mean of each data set was compared with the AAV_EGFP ischemia data using Dunnett’s test for multiple comparisons. A significance level is indicated by *P < 0.05. Each data point per biological replicate (n = 3–4) is represented by a dot in the graph. Uncropped blots are summarized in Additional file 1: Fig. S6. **I** Immunolabeling with an antibody against C3d (labeling C3, C3b, iC3b, C3dg and C3d) showed co-staining with the Müller cell marker glutamine synthetase (GLUL) in the GCL of the ischemic retina. Application of AAV_FH1-4^19-20 reduced C3 intensity at 3 dpi compared with AAV_EGFP, whereas treatment with AAV_FH1-7^19-20 resulted in a reduction after 14 dpi. Each data point per biological replicate is represented by a dot in the graph. Unpaired t-test: *P < 0.05; **P < 0.01; ***P < 0.001

FH exerts a dual regulatory effect on the central complement factor C3, as shown in Figs. 1 and 6A. First, it accelerates the decay of the C3-convertase, leading to the displacement of FB/Bb and arresting the cleavage of C3 cleavage into C3b. Second, FH serves as a cofactor for FI and facilitates the conversion of C3b into its inactive cleavage fragments: iC3b, C3dg, and C3d (Figs. 1A, 6A). After ischemia/reperfusion, a change in the C3 cleavage pattern was observed compared with control retinas at 3 dpi (Fig. 6B, Additional file 1: Fig. S5, S6) but not at 14 dpi (Additional file 1: Fig. S7). This pattern after 3 dpi was modulated by gene addition therapy with the truncated FH variants AAV_FH1-4^19-20 and AAV_FH1-7^19-20 (Fig. 6B–H). Ischemic retinas had lower levels of intact, uncleaved C3ɑ chain compared with control retinas (Fig. 6C), suggesting that consumption of full-length C3ɑ chain is related to complement activation. Nevertheless, the level of activated C3b remained comparable in ischemic and non-ischemic samples (Fig. 6D, E). The increased ratio of C3b to C3 was a characteristic feature of ischemic retinas compared to untreated retinas (Fig. 6E). This was highlighted by a markedly increased amount of C3b/C3 fragments in AAV_EGFP-treated ischemic retinas compared with AAV_EGFP-treated control retinas, indicating complement activation in the ischemic retinas. In contrast, C3b/C3 levels in AAV_FH treated eyes of ischemic retinas were more similar to those of control retinas (Fig. 6E). These observations suggest that complement activation in the ischemic retinas was attenuated by increased FH-based regulatory activity introduced by the truncated FH variants AAV_FH1-4^19-20 and AAV_FH1-7^19-20, e.g. promoting the degradation and elimination of C3b fragments (Fig. 6F–H).

The peak concentration of iC3b, C3d and C3d was detected in the ischemic retinas treated with the AAV_EGFP control vector (Fig. 6F–H). In contrast, ischemic retinas treated with vectors expressing FH1-4^19-20 and FH1-7^19-20 had iC3b, C3dg, and C3d levels lower than in the ischemic AAV_EGFP control vector treated retinas. These results emphasize that both the decay acceleration function and the cofactor activity of the truncated FH derivates were functional in this model of retinal degeneration.

Immunolabeling of C3 showed co-localization with the Müller cell marker glutamine synthetase within the GCL. Quantitative intensity assessments showed that ischemic eyes had up to 20-fold higher levels than in their non-ischemic counterparts. Remarkably, treatment with AAV_FH1-4^19-20 showed the most marked reduction at 3 dpi, with intensity scores approximately four times lower than AAV_EGFP controls (Fig. 6I). Compared with intensity values at 3 dpi, the AAV_FH1-4^19-20 treatment showed an increase by day 14 dpi. Conversely, a decrease was observed in the AAV_FH1-7^19-20 treatment group during the same period.

### Microglial/macrophage reactivity decreased after AAV_FH supplementation

**Fig. 7:**
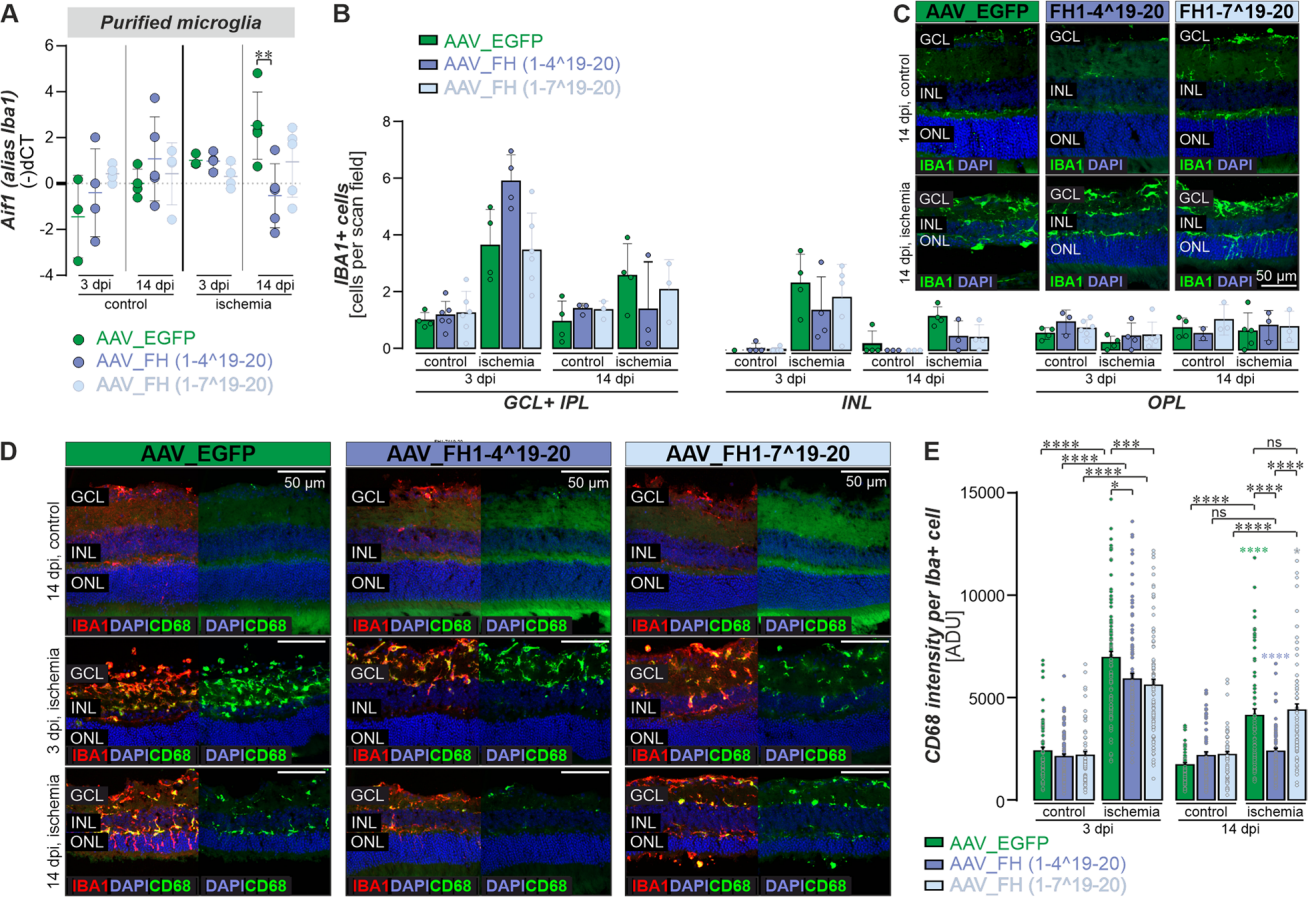
Microglia response to AAV_FH treatments following ischemia. **A** The expression of *Aif1* mRNA (also known as IBA1) in control and ischemic retinas after AAV treatment was determined by qPCR. At 14 dpi, *Aif1* levels were lower in AAV_FH1-4^19-20 treated retinas compared with AAV_EGFP ischemic controls (Unpaired t-test: **P < 0.01). **B** Quantification of IBA1-positive microglia/macrophages was performed in the respective retinal layers to detect cellular migration in response to the tissue damage (unpaired t-test). **C** Representative microscopic images of IBA1 immunostaining on retinal sections at 14 dpi. **D** Representative microscopic images of IBA1 and CD68 co-immunostaining on retinal sections at 14 dpi. **E** Mean fluorescence intensities of CD68, a lysosomal marker upregulated in activated microglia/macrophages, were measured in IBA1 + cells. Ordinary One-Way-Anova with Tukey’s multiple comparison: *P < 0.05; ***P < 0.001; ****P 0.0001. Colored asterisks indicate comparison of cells from ischemic retinas at 14 dpi versus 3 dpi of the same treatment group (as indicated by the color of the asterisk). Each data point per microglial cell (from 3–4 retinas per group) is represented by a dot in the graph. **A, B** Each data point per biological replicate (n = 3–4) is represented by a dot in the graph

The expression of allograft inflammatory factor 1 (*Aif1 transcript*) also known as ionized calcium-binding adapter molecule 1 (IBA1 protein) in immunoreactive microglia/macrophages increased sharply and peaks approximately 3 days after ischemic injury before decreasing after 7 days [26]. Our study showed that at 14 dpi, the mRNA levels of *Aif1* were significantly lower in AAV_FH1-4^19-20 treated animals compared with the AAV_EGFP control (Fig. 7A).

In the ischemic retina, we observed increased numbers of IBA1-positive cells in all retinal layers compared with the non-ischemic control. By 14 dpi, these numbers converged to the non-ischemic level. Interestingly, the number of IBA1-positive cells in AAV_FH1-4^19-20 treated eyes was 1.5-fold higher in the GCL than in the corresponding AAV_EGFP control at 3 dpi (Fig. 7B, C).

CD68 serves as an additional marker for microglial that is strongly upregulated during inflammation [27, 54, 58]. It is indicative of phagocytic activity and is upregulated in response to pro-inflammatory stimuli linked to conditions like ischemia and aging where CD68 is also associated with lipofuscin accumulation in microglia [33]. In our study, we observed a significant increase in this lysosomal protein specifically in IBA-positive microglia/macrophages in all ischemic eyes 3 dpi (Fig. 7D, E). Notably, in retinas treated with AAV_FH1-4^19-20, CD68 levels returned to baseline by 14 dpi. While there was also a decrease in CD68 levels in the other groups (EGFP and FH1-7^19-20), this reduction was not as pronounced (Fig. 7D, E).

## Discussion

In this study, we elucidated the complement regulatory potential of secreted, truncated variants of human FH in an ischemic/reperfusion model in the murine retina. Mukay et al. highlighted the importance of the complement system also in maintaining retinal health during aging [42]. They pointed to detectable retinal thinning and reduced electroretinogram amplitudes in several complement knockout strains, including C3^−/−^. Proper complement regulation by *Cfh* has also been investigated in several knockout studies, where *Cfh* was found to be central for retinal development and prevention of AMD-like pathologies [61]. Retinal development was delayed, but, while the retina appeared morphologically normal, photoreceptor and RPE cells of *Cfh*^−/−^ retinas in mice showed mitochondrial dysfunction [61]. Importantly, *Cfh*^−/−^ mice developed an AMD-like phenotype with increasing age, which could be rescued by the addition of full-length human *CFH* [14].

Here, key functional domains of the human complement regulator FH, in particular either CCP1-4^19-20 or CCP1-7^19-20, have been synthesized to develop compact but efficient complement regulators that can be encapsulated into an AAV vector. By integrating a glia-specific promoter and using an AAV capsid with a preference for Müller cells [31], we were able to target the synthesis and subsequent release of these complement regulators predominantly to Müller glia.

Our results showed that several key properties of FH1-4^19-20, as described by Schmidt et al. [57], and the novel FH1-7^19-20 variant were conserved. This occurred despite the change in expression system from yeast to mammalian cells, despite codon optimization, and despite the incorporation of a signal peptide and an epitope tag. Importantly, both variants retained a major function of FH, which is primarily determined by the CCP1-4 domains: inhibition of FB/Bb binding to C3b, resulting in accelerated C3 convertase-mediated decay. This observation is consistent with the results of other published miniFH variants [57]. A pivotal deviation was observed in heparin binding after purification of FH1-4^19-20 from HEK293 cell supernatant. Because protein domains 19 and 20 of FH are central to GAG binding, the added c-terminal myc-tag may have potentially affected these domains. Interestingly, the FH1-7^19-20 variant exhibited a better heparin binding ability compared with FH1-4^19-20. The inclusion of the GAG-binding domain 7 in FH1-7^19-20 appeared to counteract the decreased performance of domains 19-20.

In addition, the AAV vector system was confirmed to be effective, with expression occurring in Müller cells. Intravitreal injections resulted in unambiguous EGFP detection in Müller cells, which was consistent with the observed GFAP levels. By using the truncated *Gfap* promoter, expression of the added, complement regulator could be strategically linked to Müller cell gliosis. Müller cells span the whole retina and their status as one of the most prolific secretors among all retinal cell types highlights the critical role they play. These cells not only support the neurons of the retina, but also strengthen the cells of the retinal pigment epithelium, and their secretions are even detectable in the vitreous. The linkage of the AAV transcript expression and the Müller cell gliosis associated *Gfap* promotor is likely to attenuate sustained inhibition of homeostatic complement functions.

Our decision to initiate therapy post-damage aligns with potential future clinical applications, where gene therapy typically follows diagnosis. This approach poses a challenge in demonstrating therapeutic potential, given the initially low transgene expression at 3 dpi compared to 14 dpi. Morphometric analysis of the central retina revealed a strong degeneration in the ischemic eye compared to the sham injected contralateral eye. All retinal layers suffered severe cell loss, retinal thickness was reduced, and microglia became active. FH1-7^19-20 had a significant positive effect on INL cell survival, and IPL thickness was consistently higher. Similar trends, although not reaching significance, were observed for FH1-4^19-20. In AAV_EGFP-treated eyes, we detected a clear microglial activation with elevated CD68 levels after ischemia compared to non-ischemic controls. Microglia in FH1-4^19-20 mice showed control levels of CD68 14 days after ischemia.

Finally, we investigated how our treatment approach affected the transcript levels of key endogenously expressed complement components early on after ischemia/reperfusion (3 dpi) and after the tissue resettled after tissue damage (14 dpi). *C3* was less upregulated in microglia and neurons of the ischemic retina at 3 dpi when treated with the FH variants compared to EGFP controls, with FH1-7^19-20 showing the most significant effects. This could be due to a reduced demand for C3 as C3 convertases are inactivated by FH or by increased C3-fragment deposition, due to enhanced FH/FI mediated proteolytic degradation. This is supported by the lower C3b/C3 fragment detection in FH-treated retinas as detected via Western blot. The increased expression of *Cfi* transcripts in Müller cells and microglia, combined with a less pronounced upregulation of *C3* in the injured retina after FH variant treatment, and the overall downregulation of endogenous *Cfh* transcripts support the concept of our treatment approach that secreted truncated FH could modulate the local complement expression. Importantly, both truncated FH variants had a clear impact on the retinal complement homeostasis at functional level—their overexpression in Müller cells in the ischemic retina was sufficient to lower C3b/C3 protein ratios and the accumulation of C3d. Both findings suggest a reduced pro-inflammatory complement activity as the turnover of C3b to iC3b and downstream C3 fragments was enhanced [12, 23]. This also implies that overexpression of FH variants enhanced FI-mediated C3b degradation. Since we previously reported a downregulation of retinal *Cfh* mRNA after ischemia, our present results suggest that FH acts as a limiting factor for beneficial FI-mediated C3b degradation [49].

We assessed the response pattern of different retinal cell types to ischemic retinal stress by examining the gliosis response of Müller cells. Although they expressed the therapeutic transgene, we did not observe a major difference between the control and the injured retina. Both *Gfap* transcripts and protein, as determined by immunolabeling, were upregulated with a similar time course in FH-treated eyes compared to EGFP controls. The same is true for glutamine synthetase transcripts, which followed the reported pattern of an initial down-regulation immediately after ischemia with a later return to baseline levels [65]. The microglia marker *Aif1* is upregulated in this cell population with a peak around 3 days after retinal ischemia/reperfusion injury, and mRNA levels decline by day 7 after injury [26]. Here we found that after 14 days, *Aif1* mRNA levels from microglia or retinas treated with the FH1-4^19-20 variant were significantly lower than those of the corresponding AAV_EGFP-treated control. Interestingly, microglia from FH-treated retina also appeared to accumulate FH. Unfortunately, the myc-tag could not be detected by IHC staining, so it remains unclear whether these FH signals originate from the respective human truncated FH variant or the endogenously expressed murine *Cfh*. However, binding of FH to microglial surfaces has been reported in an environment of complement activation [16]. It was hypothesized that binding of FH to CR3 (CD11b) inhibits the leukocyte surface antigen CD47, leading to retention of microglia in their activated state—an effect that is stronger with the Y402H variant of FH [8]. In contrast, others found that increased FH mediated an increase in APOE binding to CR3 of monocytes, which was linked with an increase in cholesterol efflux and a decrease in the transcription of proinflammatory and proatherogenic factors [45]. This discrepancy underscores the necessity for further investigation into FH’s roles beyond complement cascade inhibition. Our own study, focusing on the microglial response to ischemia, contradicted the idea of FH-induced microglial activation. Instead, we observed a trend towards reduced microglial presence and activity in FH-treated, post-ischemic retinas compared to EGFP controls. Therefore, future research should aim to elucidate the precise ways FH influences retinal microglial responses.

### Limitations of this study

Although our expression constructs and vectors worked well, potential options for optimization should be considered. First, the sequences were codon optimized to increase expression levels. While it has been reported that adaptation of rare and slowly translated codons to more common ones in the host organism can increase expression, it is also debated whether slower translation is critical for proper peptide folding [21]. In a recent study of human *CFI* expression in mouse tissue, expression levels of codon-optimized sequences were significantly lower than the non-optimized sequence supporting the notion that codon-optimization could decrease instead of increase overall protein levels [15]. Second, to further improve Müller cell-specificity of AAV-mediated transduction, the Y445F variant of the ShH10 capsid could be considered, as it has been shown to hit as much as 50% of all Müller cells [69], whereas the original ShH10 capsid used here was only able to transduce 22% of Müller cells upon intravitreal injection [31]. Alternatively, other AAV serotypes optimized for even higher transduction efficiency, such as recombinant AAV2 serotype AAV2.GL or AAV2.NN, could be considered since cell type-specific expression of the transgene is ensured by the GFAP promoter [50].

Because AAVs are not associated with any known diseases, do not integrate efficiently into the genome, and remain in an episomal state, they are considered safer than other viral vectors [20]**.** However, as AAV vectors have recently been shown to induce immunological reactions to the virus and to contaminants in viral preparations such as DNA and peptides, this assumption has become increasingly controversial [7, 63]. AAVs were purified by polyethylene glycol precipitation and an iodixanol gradient. DNA carryover was prevented by DNAse I treatment before purification and purity was checked by gel electrophoresis. Despite all the measures taken, contamination and immunogenicity of the AAV preparations used here cannot be completely excluded. As mentioned above, some analyses of AAV_FH1-7^19-20 such as GFAP levels or transcriptional effects as well as for AAV_FH1-4^19-20 IBA1-positive microglia were unexpectedly pro-inflammatory at first glance and could be attributed to potential carry-over of contaminants. 

The ischemia/reperfusion model was used to generate an environment of acute degeneration that allowed monitoring complement activity and its response to truncated FH overexpression [49]. Following ischemic injury, secreted FH1-4^19-20 and FH1-7^19-20 demonstrated effective cofactor activity at the molecular level, resulting in the degradation and downregulation of C3b, which helped maintain cell survival and tissue integrity. Although this study did not conclusively observe these effects due to variability typically associated with the ischemia/reperfusion model [22] and limited sample sizes, further research could reveal more nuanced impacts. While our ischemia/reperfusion model was effective for this proof-of-concept study, demonstrating the functionality and complement regulatory efficacy of our approach, more pronounced treatment effects might be observed in retinal degeneration models with slower disease progression, such as retinitis pigmentosa, AMD, or diabetic retinopathy.

## Conclusion

We validated an approach to modulate local retinal complement homeostasis to improve cell survival in pathologically stressed tissue. Both FH variants showed remarkable efficacy as early as 3 days after ischemia and AAV administration, as evidenced by (i) C3b/C3 ratios and C3d fragment levels comparable to those in non-ischemic controls, (ii) a reduced number of CD68-positive, thus activated, but increased number of FH-positive microglial cells, and, (iii) a modulated cell-type-specific complement expression pattern. Our data suggest that significant beneficial effects were observed even before reaching maximum transgene expression levels, indicating that the therapy can exert its beneficial impacts shortly after administration. Our gene addition approach could benefit a broad spectrum of patients with retinal pathologies not amenable to classical gene correction approaches, such as multifactorial retinal diseases like AMD, diabetic retinopathy, or glaucoma. By specifically targeting excessive complement responses largely sparing its homeostatic functions, our approach offers a distinct advantage over existing drugs like eculizumab, pegcetacoplan, or avacincaptad pegol, which indiscriminately inhibit all complement activity. Further testing in disease-specific models and optimization of the vector system are needed to enhance efficacy, along with a deeper understanding of vector immunity.

## Methods and materials

### Animals

Adult (3–8 months of age) C57BL/6 J mice were bred in a pathogen-free animal facility following federal guidelines. All experiments were performed in accordance with European Community Council Directive 86/609/EEC and were approved by local authorities (ROB-55.2-2532.Vet_02_19_151). Animals had free access to water and food in a climate-controlled room with a 12-h light–dark cycle.

### Vector generation

FH1-4^19-20 was adapted with permission from the miniFH developed by Schmidt et al. to express the gene in a murine system a signal peptide and a c-terminal myc tag were added to the sequence and codons were optimized using the software tool of the company that also synthesized the final DNA (genewiz, Regensburg, Germany). A synthesized sequence of FH domains 5–7 was subcloned into the FH1-4^19-20 vector to create the FH1-7^19-20 variant. For functional tests that required large amounts of protein, genes were cloned into a CMV promoter expression plasmid (Thermo Fisher, pcDNA3.1). For AAV production, the expression cassette containing the gfABC1D version of the GFAP promoter (developed by [32]), the transgenes and an IRES sequence linked GFP reporter were cloned into a transfer plasmid.

### AAV production

Recombinant AAV (rAAV) production was based on the protocol of [72]. CaPO_4_ precipitation was used to transfect HEK 293 T cells with equimolar levels of a helper plasmid, the ShH10 RepCap plasmid and the transfer plasmids containing the respective expression cassettes (Fig. 2B). After 72 h, cells and supernatant were collected separately. AAVs from the supernatant were precipitated by addition of 25 ml of 40% PEG/NaCl solution (24 g NaCl, 400 g PEG 8000 in 1 l (w/v) ddH_2_O) to 100 ml of supernatant. The solution was stirred for 1 h at 4 °C, then kept at 4 °C without stirring and finally precipitates were spun down at 2818 × g for 15 min. Collected cells were pelleted in the same manner and resuspended in lysis buffer. PEG precipitated virus and BitNuclease (250 units/u, Biotool Co, Houston, TX, B16003) were added, and the solution was incubated 2 h at 37 °C. Samples were frozen in liquid nitrogen and thawed. Cell debris was removed by centrifuging 8000 × g 30 min. Virus containing supernatants were purified by iodixanol gradient ultracentrifugation (15, 25, 40, and 56% iodixanol 50,000 × g for 2 h 17 min at 22 °C in a Ti70 rotor (Beckman, Fullerton, CA, USA)). AAV containing 40% iodixanol fraction was collected with a syringe and dialyzed using a Slide-A-Lyzer 10000 MWCO 5 ml (Thermo Scientific, 66380). Buffer change to PBS with 0.001% F68 and concentration of the virus was performed with Vivaspin 6 (Sartorius, Göttingen, Germany, Cat. VS0602). AAVs were quantified by Sybr Green qPCR with SV40 primers (D’Costaet al, 2016). 1e10 genomic copies of AAVs in 1 µl of diluent were injected into the vitreous of each eye if not stated otherwise.

### Ischemia and AAV injection

The murine ischemia model is a well described procedure to introduce retinal degeneration [19]. In brief, to induce ischemia, animals were anesthetized with a ketamine/xylazine cocktail (100 mg/kg body weight and 5 mg/kg body weight respectively), and the anterior ocular chamber was punctured with a 30-gauge cannula connected to an isotonic saline drip. The reservoir of the drip was raised 2 m above the animal to create a pressure (160 mmHg) higher than the systolic pressure of the mouse (< 130 mm Hg), thus occluding retinal arteries. After 60 min, the drip was removed and 1 µl of AAV solution was injected intravitreally into both eyes.

### Purification of murine retinal cell types

As previously reported in detail by [19], retinal cell types were isolated from murine retinas and sorted into cell fractions with the Miltenyi magnetic cell sorting system. Retinas were immediately removed from enucleated eyes and digested in 12 mM PBS glucose containing 0.2 mg/ml papain (Roche). For 30 min 37 °C retinas were washed and treated with Dnase I in PBS/Glucose (200 U/ml, 4 min, room temperature). Supernatant was replaced with an extracellular solution (136 mM NaCl, 3 mM KCl, 10 mM HEPES, 11 mM glucose, 1 mM MgCl_2_ and 2 mM CaCl_2_, pH 7.4) and the tissue dissociated. Cell types were subsequently subtracted from the cell suspension by incubating it with specific antibodies coupled to magnetic microbeads (CD11b for microglia, CD31 for perivascular cells and CD29 for Müller cells (Milteny Biotec)). In brief, for every purification step, the suspension was pipetted onto and large cell column (Milteny Biotec, Cat. 130-042-202), clipped to a magnetic rack. Non-magnetically labeled cells were eluted by washing. The column was removed from the rack and labeled cells were eluted, spun down (10,000 × g, 15 min, 4 °C) and the pellet was immediately frozen in dry ice. Finally, the neuron rich cell suspension depleted of microglia, vascular and Müller cells was collected in the same manner.

### qPCR complement transcript analysis

PureLink RNAMicro Kit (Invitrogen, Cat. 2183–016) was used according to the manufacturers specifications to extract RNA from cell fractions. RNA was eluted in 10 µl RNAse free water and transcribed into cDNA with the RevertAid First Strand cDNA Synthesis Kit (ThermoFischer, Cat. K1621) using random hexamer primers. All cDNA was diluted 1:4 in nuclease free water. A 384 well plate was filled with 2.5 µl of silicone oil in each well to prevent evaporation during qPCR cycles. 1 µl of diluted cDNA as well as 1.5 µl of TaqMan™ Fast Advanced Master Mix (Thermo Fisher, Cat TF4444556) containing assay specific primers (Table 1, Metabion International, Planegg, Germany) and TaqMan probes (Roche) were added. For complement component analysis, commercial qPCR by Thermo fisher were multiplexed with a primer limited *pdhb* housekeeper assay (Mm00437859_g1, Mm00442739_m1, Mm01341415_m1, Mm01143935_g1, Mm00432470_m1, Mm01132441_g1, Mm00499323_m1). Final pipetting into the plate wells was done with a non-contact liquid handler (i.dot). qPCR assays were measured with a QuantStudio 6 machine (Thermo Fisher).

**Table 1:** Primers used for qPCR analysis

Target gene	Oligo	Probe
TM_EGFP_for	cgaccactaccagcagaaca	Nr. 74
TM_EGFP_rev	tctcgttggggtctttgc	
TaqMan_GFAP_for	tcgagatcgccacctacag	Nr. 67
TaqMan_GFAP_rev	gtctgtacaggaatggtgatgc	
TaqMan_Nrl_202_for	tgcctttctggttctgacagt	Nr. 53
TaqMan_Nrl_202_rev	gaaagccattctgggactga	
TaqMan_CD29_rev	cacaacagctgcttctaaaattg	Nr. 41
TaqMan_CD29_for	tccataaggtagtagagatcaataggg	
TaqMan_Nrl_202_for	tgcctttctggttctgacagt	Nr. 53
TaqMan_Nrl_202_rev	gaaagccattctgggactga	
TM_maus_Pecam1_for	gctggtgctctatgcaagc	Nr. 64
TM_maus_Pecam1_rev	atggatgctgttgatggtga	
humanFH_Variant_for	GCCAGCTCTGTGGAATACCA	Nr. 97
humanFH_Variant_rev	ACTGGCCGTTTCTACAGGTG	

### Western blot analysis

The retinas from mouse eyes were isolated and suspended in T-PER buffer supplemented with protease and phosphatase inhibitors. Protein isolation was achieved by sonicating the samples for 10 min and incubating them for 1 h on a shaker at 4 °C. Denatured proteins from the retina, RPE/choroid, or serum were separated in reducing Laemmli buffer on a 12%, SDS-PAGE and transferred to PVDF membranes. Membranes were blocked for 2 h in blocking buffer (5% skim milk in TBST) before being incubated with the appropriate primary antibody in blocking buffer overnight at 4 °C. Following washing, membranes were incubated for 2 h at room temperature in blocking buffer with appropriate HRP secondary antibodies. Following a final washing step, membranes were developed with lumi-light blotting substrate or WesternSure PREMIUM Chemiluminescent Substrate (Licor, Bad Homburg, Germany).

### C3b assay

For ELISA based quantification of C3b, 96 well polysorp flat-bottom plates were coated with 10 µg/ml lipopolysaccharide from Salmonella typhosa (L6386, Sigma Aldrich/Merck, Darmstadt, Germany) in PBS and incubated overnight at 4 °C. Normal mouse serum was diluted 1:10 and incubated with activation buffer containing various concentrations of FHs variants. Following a 1 h incubation at 37 °C, samples were washed and a secondary antibody C3-HRP (Table 3) was used for development.

### Immunohistochemistry

Mouse eyes were fixed in 4% PFA for 1 h, incubated in sucrose (30% w/v in PBS) for cryoprotection, embedded in OCT compound and cut into sections of 20 μm thickness using a cryostat. Retinal slices were permeabilized (0.2% triton X-100 in PBS) for ten minutes and then stained with primary antibodies (Table 2) diluted in blocking buffer (0.1% tween and either 3% normal donkey serum or 5% normal goat serum in PBS, respective of the secondary antibody used) overnight at 4 °C. The slides were then incubated in a dilution of their respective secondary antibodies (Table 3) and 4,6-diamidino-2-phenylindole (DAPI, Sigma-Aldrich, 1:1000) in an identical blocking buffer for 45 min at room temperature, mounted using Aqua-Poly/Mount (Polysciences, Warrington, PA, USA) and dried overnight. Experiments missing primary antibody incubation served as negative controls. The samples were scanned using a confocal microscope (custom-made VisiScope CSU-X1 confocal system equipped with high-resolution sCMOS camera; Visitron Systems, Puchheim, Germany).

**Table 2:** Primary antibodies used in the present study

Primary antibodies	Host	Dilution	Source	Catalogue number
GFAP	Mouse	1:500	Sigma Aldrich/Merck (Darmstadt, Germany)	G3893
C3d	Goat	2 µg/ml	R&D Systems (Minneapolis, MN, USA)	AF2655
EGFP	Goat	1:200	Rockland Immunochemicals (Limerick, PA, USA)	600 101 215
IBA-1	Rabbit	1:500	Wako Chemicals (Neuss, Germany)	019-19741
FH	Mouse	1:1200	Santa Cruz Biotechnology (Dallas, TX, USA)	sc-166613
FH	Goat	1:500	Sigma Aldrich/Merck (Darmstadt, Germany)	341276
Calretinin	Goat	1:500	Swant (Burgdorf, Switzerland)	CG1
GLUL	Mouse	1:500	Millipore	MAB302
CD68	Rat	1:500	AbDSerotec	MCA1957GA

**Table 3:** Secondary antibodies used in the present study

Secondary antibodies	Host	Dilution	Source	Catalog number
Alexa Fluor 647 anti-mouse	Donkey	1:500	Life Technologies/ Thermo Fisher Scientific (Waltham, MA, USA)	A31571
Alexa Fluor 647 anti-mouse-IgG1	Goat	1:500	Invitrogen (Massachusetts, USA)	A21240
Alexa Fluor 647 anti-goat	Donkey	1:500	Dianova (Hamburg, Germany)	705-605-003
Cy3 anti-goat	Donkey	1:500	Dianova (Hamburg, Germany)	705-165-147
Cy2 anti-goat	Donkey	1:500	Dianova (Hamburg, Germany)	705-225-147
Cy5 anti-rabbit	Donkey	1:500	Dianova (Hamburg, Germany)	711-175-152
Cy3 anti-rabbit	Goat	1:500	Dianova (Hamburg, Germany)	111-165-144
Alexa Fluor 488 anti-rat	Donkey	1:500	Life Technologies/ Thermo Fisher Scientific (Waltham, MA, USA)	A21208

Apoptotic and necrotic retinal cells were identified via terminal deoxynucleotidyl transferase dUTP Nick End Labeling (TUNEL) assay (In Situ Cell Death Detection Kit, TMR red by Roche Molecular Biochemicals, Mannheim, Germany) following the manufacturer’s instruction and were imaged imaged using the VisiScope CSU-X1 confocal system.

Retinal flat mounts were carefully removed from fixed eye cups and were then subjected as free floating samples to the staining procedure. After 2 h of permeabilization (0.2% triton X-100 in PBS), they were stained with primary antibodies (Table 2) diluted in blocking buffer (0.1% tween and either 3% normal donkey serum or 5% normal goat serum in PBS, respective of the secondary antibody used) four 48 h at 4 °C. Flat mounts were then incubated in a dilution of their respective secondary antibodies (Table 3) and 4,6-diamidino-2-phenylindole (DAPI, Sigma-Aldrich, 1:1000) in an identical blocking buffer for 24 h at room temperature, mounted using Aqua-Poly/Mount (Polysciences, Warrington, PA, USA) and dried overnight. Tile scans (9 z levels per flat mount with evenly distributed spacing between 9 and 11 µm) of whole retinal flat mounts were performed with the Zeiss Axio Imager 2 equipped with Axiocam506 mono using ZEN software (Zeiss, Oberkochen). For detailed co-localization studies central areas of the flat mounts were imaged using the VisiScope CSU-X1 confocal system. Detailed analysis of images including the assessment of morphometric parameters were assessed using Fiji [55].

### Statistical analysis

The data were analyzed with GraphPad PRISM 7 (version 7.03) and reported as mean ± standard error (SEM). Unless otherwise stated, statistical testing was limited to the unpaired t-test due to the number of biological replicates available and the moderate magnitude of effects. Identification of outliers was also performed with GraphPad PRISM 7 (ROUT, Q = 1%).

### Supplementary Information

**Additional file 1.** Supplementary figures an data tables.

## Data Availability

All data supporting the results of this study are available in the article and its supplementary information.
