# Supplementary material for: Gliosis-dependent expression of complement factor H truncated variants attenuates retinal neurodegeneration following ischemic injury
Source: J Neuroinflammation. 2024 Feb 22;21:56. doi: 10.1186/s12974-024-03045-3 (PMC10885619; doi:10.1186/s12974-024-03045-3)
Supplement: Supplementary file 1 — Additional file 1. Supplementary figures an data tables. [file 12974_2024_3045_MOESM1_ESM.pdf]

Supplemental information

Gliosis dependent expression of complement factor H truncated variants attenuates retinal neurodegeneration following ischemic injury

Josef Biber[1#], Yassin Jabri[2#], Sarah Glänzer[1], Aaron Dort[3], Patricia Hoffelner[1], Christoph Q. Schmidt[4], Oliver Bludau[1], Diana Pauly[3#*], Antje Grosche[1#]

[1]Department of Physiological Genomics, Ludwig-Maximilians-Universität München, Planegg-Martinsried, Germany

[2]Department of Ophthalmology, University Hospital Regensburg, Regensburg, Germany

[3]Experimental Ophthalmology, University of Marburg, Marburg, Germany

[4]Institute of Experimental and Clinical Pharmacology, Toxicology and Pharmacology of Natural Products, University of Ulm Medical Center, Ulm, Germany

[5]Institute of Pharmacy, Biochemical Pharmacy Group, Martin Luther University Halle-Wittenberg, Halle, Germany Halle, Germany

[#]contributed equally

*corresponding author: diana.pauly@uni-marburg.de

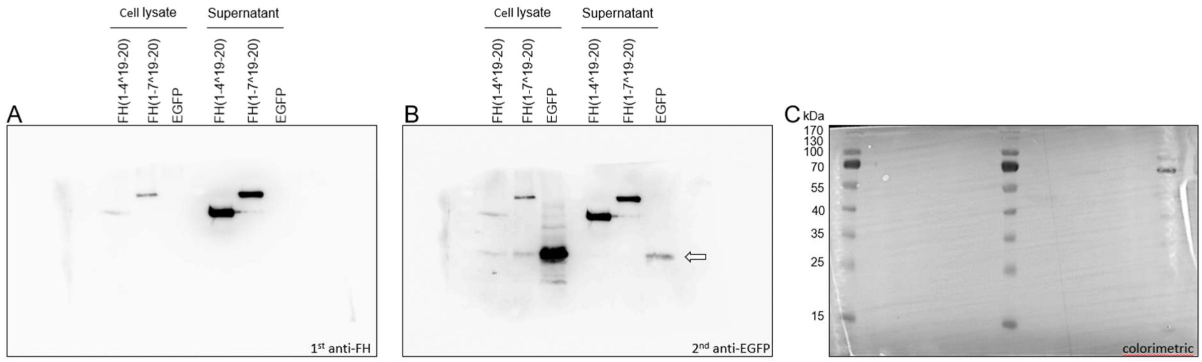

Figure S1. Original documented uncropped blot for Figure 1D.

A First, blot incubated with anti-FH and HRP-secondary antibody. Detection was performed with chemiluminescent substrate.

B Second, blot of A was incubated with anti-EGFP and HRP-secondary antibody. Detection was performed with chemiluminescent substrate. Additional anti-EGFP specific signal is marked with an arrow.

C Blot shown with pre-stained, colorimetric molecular weight marker.

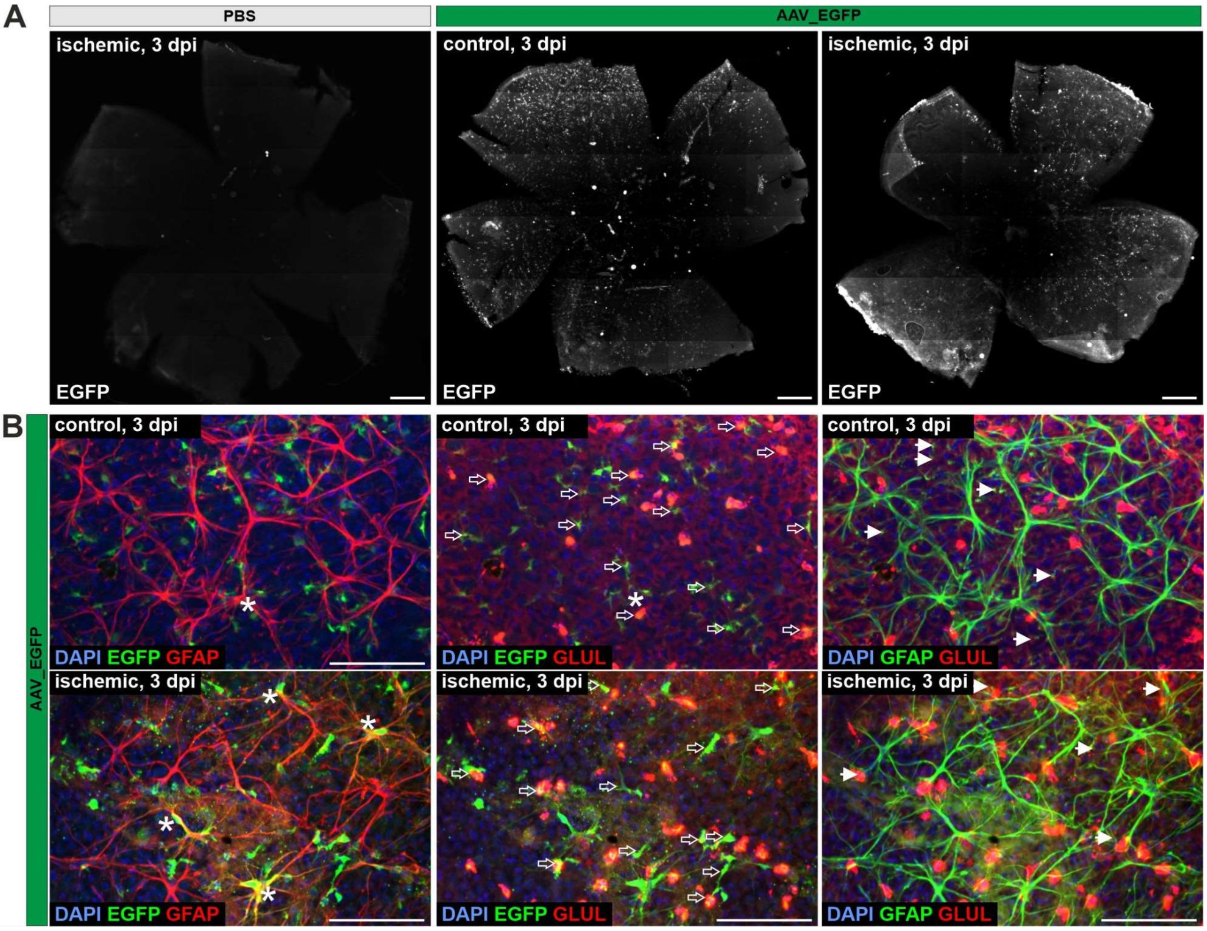

Figure S2. Confirmation of transgene expression as early as 3 dpi upon intravitreal injection of AAV_EGFP.

A Tile scans of retinal flat mounts collected and fixed at 3 dpi. EGFP fluorescence was enhanced by antibody labeling for EGFP using an Alexa488-conjugated secondary antibody. Scale bars, 500 µm.

B Representative images of retinal flat mounts focused on the nerve fiber/ganglion cell layer at 3 dpi after AAV_EGFP delivery. Co-labeling was performed to examine EGFP expression in astrocytes and Müller cells, the two major retinal macroglial cell types.
Top row, in non-ischemic control eyes, only a few GFAP+ astrocytes, identified by their location in the tissue and their star-shaped morphology, also showed EGFP expression (asterisk). In contrast, many GLUL-positive Müller cells show EGFP expression. Overlay of GLUL and GFAP shows that Müller cells upregulated GFAP already in the non-ischemic control eye, most likely in response to the intravitreal injection of AAV.
Bottom row, in the post-ischemic retina many more astrocytes appear EGFP positive, while also the GFAP+GLUL+ Müller cells show transgene expression. Scale bars, 100 µm.

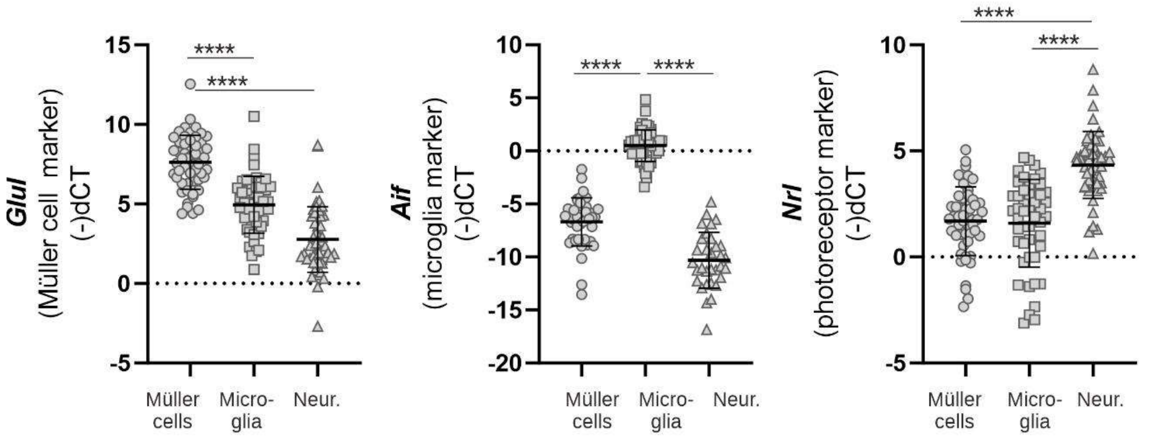

Figure S3. Validation of cell purification by magnetic activated cell sorting. mRNA levels of cell markers in the respective fractions were analyzed by qPCR.

X axis = cell fractions. Analyzed with Student's t-Test (n = 58-61 animals). Note that conditions (control, post-ischemic) and genotypes were combined for this analysis.

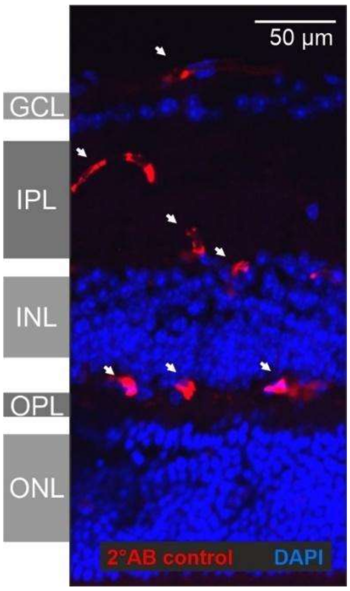

Figure S4. Secondary antibody control of immunostaining on mouse retinal tissue.

Arrows point towards blood vessels that are likely stained due to binding of the secondary anti mouse antibody against endogenous antibodies. Stained with Alexa Fluor 647 anti-mouse-IgG1 A21240, Invitrogen 1:500.

WB_221108

1X SDS-PAGE Running Buffer Bio-Rad Recipe
1. 25 mM Tris-HCl
2. 192 mM Glycin
3. 0.1 % SDS

1X Transfer Buffer
1. 25 mM Tris
2. 192 mM Glycin
3. 20 % MeOH
4. 0.1 % SDS

SDS-Page

Gel 12%, 1,5 mm, 10 combs, fastcast stain-free

80 V 25 min, 100 V 70 min

Protein Transfer

Gel 1: Immobilon-P PVDF
PowerPacBasic: 100 min, constant 385 mA, ~ 100 V

Marker
170 – 10 kDa PeqGold IV, 7 µL
250 – 10 kDa Precision Plus unstained 10 µL

Primary antibodies
In 5 % Milk/TBST

1. P173 goat anti-mouse C3d 1:1000

Secondary antibodies
In 5 % Milk/TBST

1. S84 rabbit anti-goat POD 1:5000

Samples

Loading Dye, Reduing Agent: Roti-Load 1 (reducing)

1. 22131 AAV_FH1-7^19-20, ischemia, right retina 70 µg
2. 223 AAV_FH1-7^19-20, ischemia, right retina 70 µg
3. 11308 AAV_FH1-4^19-20, ischemia, right Retina 70 µg
4. 11312 AAV_FH1-4^19-20, ischemia, right Retina 70 µg
5. 11296 AAV_EGFP, ischemia, right Retina 70 µg
6. 11298 AAV_EGFP, ischemia, right Retina 70 µg
7. 11320 AAV_FH1-7^19-20, control, left Retina 70 µg
8. 223 AAV_FH1-7^19-20, control, left Retina 70 µg
9. 11308 AAV_FH1-4^19-20, control, left Retina 70 µg
10. 11314 AAV_FH1-4^19-20, control, left Retina 70 µg
11. 11298 AAV_EGFP, control, left Retina 70 µg
12. 225 AAV_EGFP, control, left Retina 70 µg

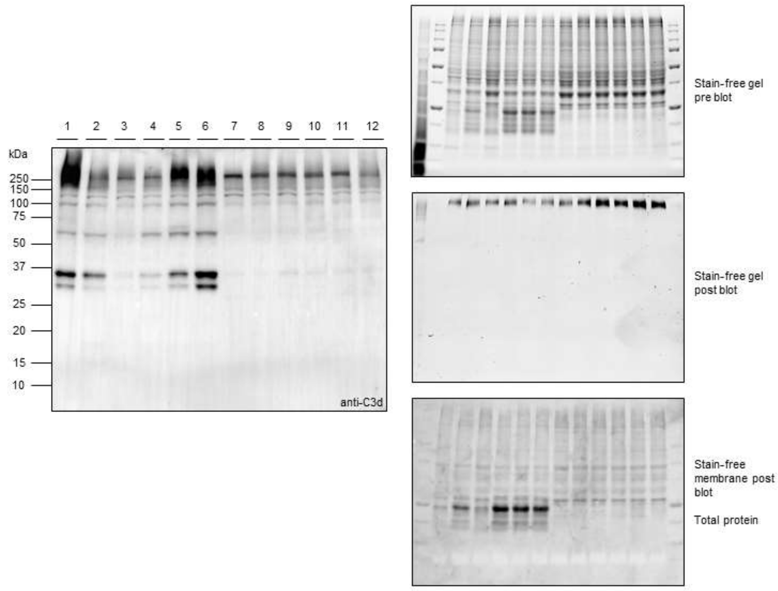

Figure S5. Original documentation for uncropped blots presented and analyzed in/for Figures 6B.

1X SDS-PAGE Running Buffer Bio-Rad Recipe | WB_221115
1. 25 mM Tris-HCl
2. 192 mM Glycin
3. 0.1 % SDS

1X Transfer Buffer
1. 25 mM Tris
2. 192 mM Glycin
3. 20 % MeOH
4. 0.1 % SDS

SDS-Page

Gel 12%, 1.5 mm, 10 combs, fastcast stain-free

80 V 25 min, 100 V 75 min

Protein Transfer

Gel 1: Immobilon-P PVDF
PowerPacBasic: 100 min, constant 340 mA, ~ 100 V

Marker
170 – 10 kDa PeqGold IV, 7 µL
250 – 10 kDa Precision Plus unstained 10 µL

Primary antibodies
In 5 % Milk/TBST

1. P173 goat anti-mouse C3d 1:1000

Secondary antibodies
In 5 % Milk/TBST

1. S84 rabbit anti-goat POD 1:5000

Samples

Loading Dye, Reduing Agent: Roti-Load 1 (reducing)

1. 22131 AAV_FH1-7^19-20, ischemia, right retina 40 µg
2. 11320 AAV_FH1-7^19-20, ischemia, right retina 40 µg
3. 22253 AAV_FH1-7^19-20, ischemia, right retina 40 µg
4. 223 AAV_FH1-7^19-20, ischemia, right retina 40 µg
5. 11308 AAV_FH1-4^19-20, ischemia, right retina 40 µg
6. 11310 AAV_FH1-4^19-20, ischemia, right retina 40 µg
7. 22131 AAV_FH1-7^19-20, control, left retina 40 µg
8. 11320 AAV_FH1-7^19-20, control, left retina 40 µg
9. 22253 AAV_FH1-7^19-20, control, left retina 40 µg
10. 233 AAV_FH1-7^19-20, control, left retina 40 µg
11. 11308 AAV_FH1-4^19-20, control, left retina 40 µg
12. 11310 AAV_FH1-4^19-20, control, left retina 40 µg

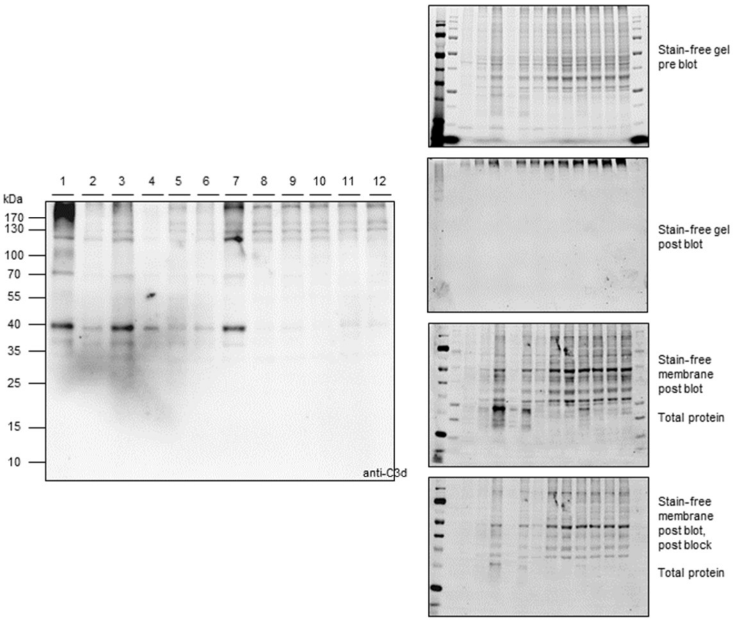

1X SDS-PAGE Running Buffer Bio-Rad Recipe
1. 25 mM Tris-HCl
2. 192 mM Glycin
3. 0.1 % SDS

1X Transfer Buffer
1. 25 mM Tris
2. 192 mM Glycin
3. 20 % MeOH
4. 0.1 % SDS

SDS-Page

Gel 12%, 1.5 mm, 10 combs, fastcast stain-free

80 V 25 min, 100 V 75 min

Protein Transfer

Gel 1: Immobilon-P PVDF
PowerPacBasic: 100 min, constant 340 mA, ~ 100 V

Marker
170 – 10 kDa PeqGold IV, 7 µL
250 – 10 kDa Precision Plus unstained 10 µL

Primary antibodies
In 5 % Milk/TBST

1. P173 goat anti-mouse C3d 1:1000

Secondary antibodies
In 5 % Milk/TBST

1. S84 rabbit anti-goat POD 1:5000

Samples

Loading Dye, Reduing Agent: Roti-Load 1 (reducing)

1. 11312 AAV_FH1-4^19-20, ischemia, right retina 40 µg
2. 11314 AAV_FH1-4^19-20, ischemia, right retina 40 µg
3. 11296 AAV_EGFP, ischemia, right retina 40 µg
4. 11298 AAV_EGFP, ischemia, right retina 40 µg
5. 11304 AAV_EGFP, ischemia, right retina 40 µg
6. 225 AAV_EGFP, ischemia, right retina 40 µg
7. 11304 AAV_EGFP, control, left retina 40 µg
8. 11296 AAV_EGFP, control, left retina 40 µg
9. 11308 AAV_FH1-4^19-20, control, left retina 40 µg
10. 11314 AAV_FH1-4^19-20, control, left retina 40 µg
11. 11298 AAV_EGFP, control, left retina 40 µg
12. 225 AAV_EGFP, control, left retina 40 µg

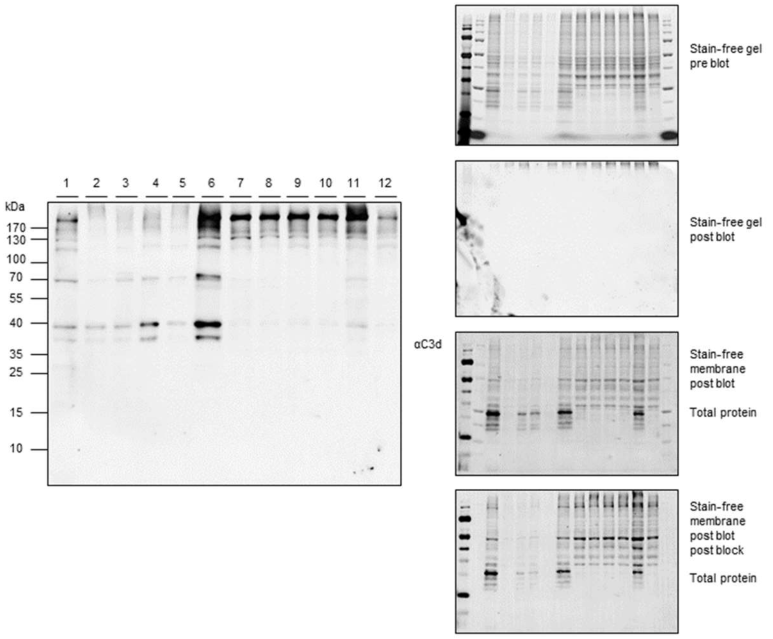

Figure S6. Original documentation for uncropped blots presented and analyzed in/for Figures 6C-H.

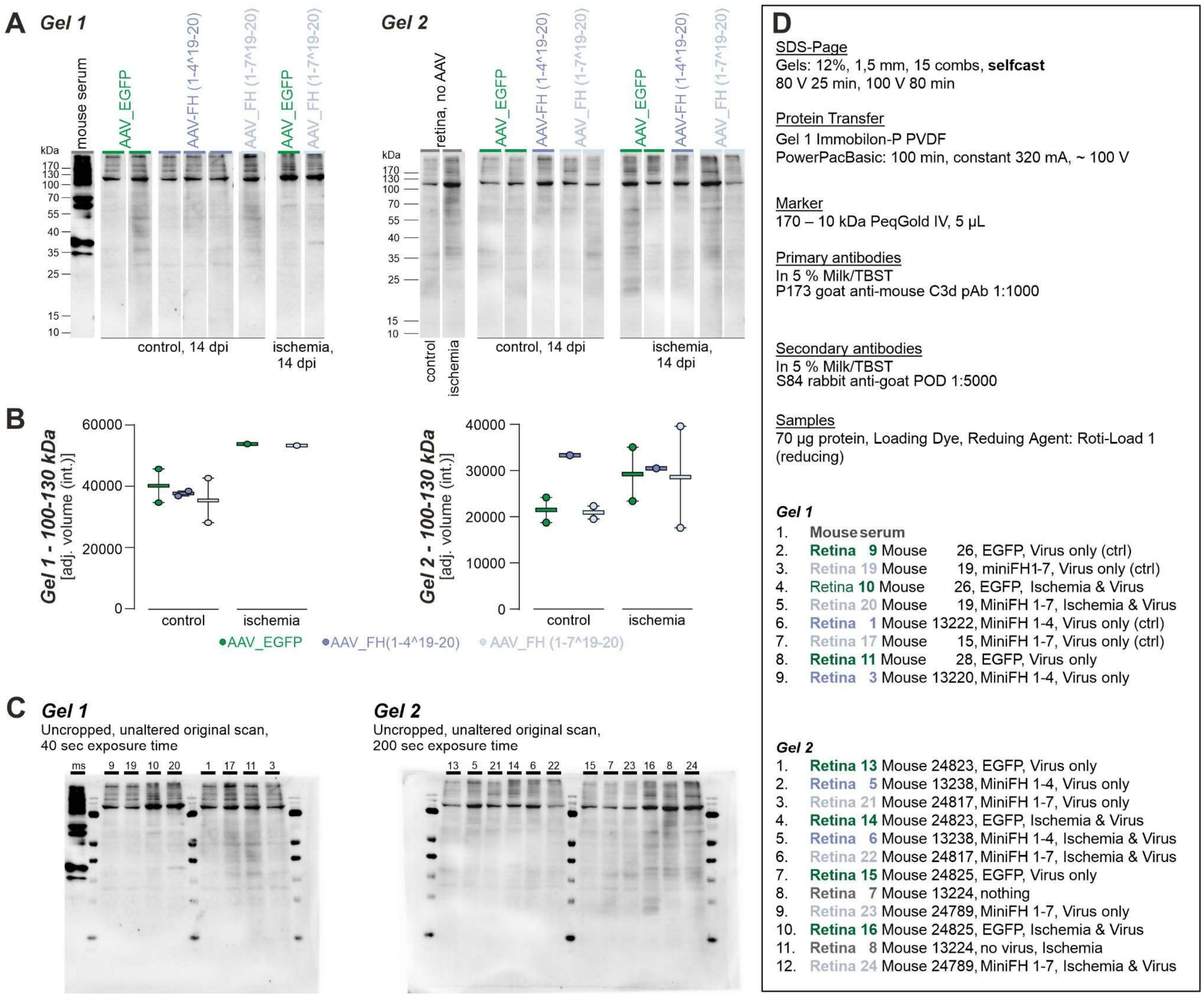

Figure S7. Original documentation for the detection of C3 in retinal lysate 14 days after AAV injection.

Two Western blots were loaded with retinal lysate protein samples (70 µg), including control and ischemic tissue treated with either AAV_EGFP, AAV_1-4^19-20 or AAV_1-7^19-20. Gel/Blot 1 was developed for 40 seconds and the Gel/Blot 2 for 200 seconds.

A After 14 days of ischemia, there were no differences in the C3 cleavage pattern between the control and ischemic groups, as well as the AAV-treated groups.

B Densitometric analysis showed a stronger C3/C3b band at 100-130 kDa for ischemic samples.

C The original unprocessed blot is shown.

Table S1. Summary of statistical analysis (unpaired t-test) of the qPCR assay for differences of AAV_EGFP injected vs AAV_FH1-4^19-20 injected samples. FC: Fold change, dct: delta CT, ddCt: delta delta CT

Fraction	Target	dpi	Eye	EGFP -dct	FH1-4^19-20 -dct	FC (ddCt)	P-value
Microglia	*C3*	3	Control	-4.3934	-2.6833	1.7101	0.2214
Microglia	*C3*	3	Ischemic	0.1402	0.0064	-0.1338	0.7965
Microglia	*C3*	14	Control	1.3203	-2.8053	-4.1256	0.1751
Microglia	*C3*	14	Ischemic	1.5964	-0.7537	-2.3501	0.0707
Microglia	*Cfb*	3	Control	-3.3269	-1.8609	1.4659	0.2488
Microglia	*Cfb*	3	Ischemic	0.9835	0.6351	-0.3485	0.7273
Microglia	*Cfb*	14	Control	-1.9110	-2.9475	-1.0365	0.5833
Microglia	*Cfb*	14	Ischemic	1.0962	-1.5012	-2.5974	0.0841
Microglia	*Cfd*	3	Control	-5.1225	-6.6463	-1.5238	0.5386
Microglia	*Cfd*	3	Ischemic	-7.3497	-5.3208	2.0289	0.4714
Microglia	*Cfd*	14	Control	-7.9227	-6.8890	1.0336	0.5526
Microglia	*Cfd*	14	Ischemic	-6.4427	-5.0212	1.4215	0.6285
Microglia	*Cfh*	3	Control	-0.8964	-0.6603	0.2360	0.7305
Microglia	*Cfh*	3	Ischemic	-0.8967	-0.6722	0.2245	0.5966
Microglia	*Cfh*	14	Control	0.2234	-0.1398	-0.3632	0.5950
Microglia	*Cfh*	14	Ischemic	1.1136	0.0302	-1.0834	0.0841
Microglia	*Cfi*	3	Control	-5.1787	-5.2475	-0.0688	0.9240
Microglia	*Cfi*	3	Ischemic	-6.6886	-7.3657	-0.6771	0.4356
Microglia	*Cfi*	14	Control	-5.9388	-4.5005	1.4383	0.3577
Microglia	*Cfi*	14	Ischemic	-5.0866	-5.0442	0.0424	0.9680
Microglia	*Cfp*	3	Control	-2.4964	-2.5089	-0.0125	0.9874
Microglia	*Cfp*	3	Ischemic	1.6709	0.1903	-1.4806	0.1313
Microglia	*Cfp*	14	Control	-2.5868	-1.7547	0.8321	0.4304
Microglia	*Cfp*	14	Ischemic	0.2704	-0.5801	-0.8505	0.4616
Müller Cells	*C3*	3	Control	-1.1192	-0.5795	0.5396	0.6008
Müller Cells	*C3*	3	Ischemic	1.1076	1.4058	0.2982	0.7229
Müller Cells	*C3*	14	Control	-0.5194	-1.0005	-0.4811	0.4357
Müller Cells	*C3*	14	Ischemic	1.8275	2.1329	0.3054	0.5329
Müller Cells	*Cfb*	3	Control	-2.9973	-2.0468	0.9504	0.2986
Müller Cells	*Cfb*	3	Ischemic	-1.0299	-1.2181	-0.1882	0.8110
Müller Cells	*Cfb*	14	Control	-3.4479	-2.7466	0.7013	0.4456
Müller Cells	*Cfb*	14	Ischemic	-1.1829	-1.3978	-0.2149	0.7947
Müller Cells	*Cfd*	3	Control	-6.5176	-3.6390	2.8786	0.1698
Müller Cells	*Cfd*	14	Ischemic	-9.0726	-8.5326	0.5400	--
Müller Cells	*Cfd*	14	Control	-8.0431	-7.5611	0.4820	0.8161
Müller Cells	*Cfd*	14	Ischemic	-8.8784	-7.3351	1.5433	0.6216
Müller Cells	*Cfh*	3	Control	-1.3348	-1.0415	0.2933	0.3285
Müller Cells	*Cfh*	3	Ischemic	-2.5357	-2.0900	0.4457	0.4079
Müller Cells	*Cfh*	14	Control	-1.1971	-0.9613	0.2358	0.4683
Müller Cells	*Cfh*	14	Ischemic	-0.6509	-0.6315	0.0193	0.9589

**Müller Cells**	***Cfi***	**3**	**Control**	-5.6757	-4.8665	0.8092	0.4644
**Müller Cells**	***Cfi***	**3**	**Ischemic**	-6.2857	-5.5857	0.7000	0.6297
**Müller Cells**	***Cfi***	**14**	**Control**	-7.6991	-6.3111	1.3880	0.4125
**Müller Cells**	***Cfi***	**14**	**Ischemic**	-6.1561	-4.8483	1.3079	**0.0449**
**Müller Cells**	***Cfp***	**3**	**Control**	-2.9230	-2.7960	0.1271	0.6726
**Müller Cells**	***Cfp***	**3**	**Ischemic**	-4.2322	-3.9520	0.2802	0.4695
**Müller Cells**	***Cfp***	**14**	**Control**	-3.1376	-2.8357	0.3019	0.3260
**Müller Cells**	***Cfp***	**14**	**Ischemic**	-3.4938	-2.7131	0.7807	0.0571
**Neurons**	***C3***	**3**	**Control**	-5.2992	-6.1707	-0.8715	0.3788
**Neurons**	***C3***	**3**	**Ischemic**	-1.1912	-3.1842	-1.9930	0.1940
**Neurons**	***C3***	**14**	**Control**	-5.9962	-7.1729	-1.1767	0.3045
**Neurons**	***C3***	**14**	**Ischemic**	-5.1828	-3.9320	1.2508	0.1959
**Neurons**	***Cfb***	**3**	**Control**	-3.5318	-3.6999	-0.1681	0.6639
**Neurons**	***Cfb***	**3**	**Ischemic**	-2.9066	-3.7141	-0.8076	0.4430
**Neurons**	***Cfb***	**14**	**Control**	-4.8694	-3.5430	1.3264	0.1662
**Neurons**	***Cfb***	**14**	**Ischemic**	-6.3055	-4.2957	2.0099	0.3687
**Neurons**	***Cfd***	**3**	**Control**	-11.3754	-10.3437	1.0316	0.4512
**Neurons**	***Cfd***	**3**	**Ischemic**		-2.0670	-2.0670	--
**Neurons**	***Cfd***	**14**	**Control**	-10.6014	-9.8430	0.7584	0.7474
**Neurons**	***Cfd***	**14**	**Ischemic**	-12.3602	-9.9296	2.4305	0.3973
**Neurons**	***Cfh***	**3**	**Control**	-4.9610	-4.0908	0.8702	0.3266
**Neurons**	***Cfh***	**3**	**Ischemic**	-4.9317	-5.2145	-0.2828	0.5013
**Neurons**	***Cfh***	**14**	**Control**	-5.5778	-5.0376	0.5401	0.4169
**Neurons**	***Cfh***	**14**	**Ischemic**	-6.8643	-6.0345	0.8298	0.2109
**Neurons**	***Cfi***	**3**	**Control**	-3.3386	-4.1305	-0.7918	0.3144
**Neurons**	***Cfi***	**3**	**Ischemic**	-3.2678	-3.1599	0.1078	0.8671
**Neurons**	***Cfi***	**14**	**Control**	-6.5665	-5.7804	0.7860	0.3672
**Neurons**	***Cfi***	**14**	**Ischemic**	-5.4552	-6.1631	-0.7079	0.5565
**Neurons**	***Cfp***	**3**	**Control**	-1.9795	-1.9820	-0.0025	0.9965
**Neurons**	***Cfp***	**3**	**Ischemic**	-2.5492	-2.3774	0.1718	0.7811
**Neurons**	***Cfp***	**14**	**Control**	-2.8764	-2.3586	0.5178	0.3448
**Neurons**	***Cfp***	**14**	**Ischemic**	-2.0179	-2.6538	-0.6358	0.6570

Table S2: Summary of statistical analysis (unpaired t-test) of the qPCR testing for differences of AAV_EGFP vs AAV_FH1-7^19-20 injected samples. FC: Fold change, dct: delta CT, ddCt: delta delta CT

Fraction	Target	dpi	Eye	EGFP -dct	FH1-7^19-20 -dct	FC (ddCt)	P-value
**Microglia**	***C3***	**3**	**Control**	-4.3934	-2.6834	1.7100	0.1596
**Microglia**	***C3***	**3**	**Ischemic**	0.1402	-1.0620	-1.2022	**0.0054**
**Microglia**	***C3***	**14**	**Control**	1.3203	0.8819	-0.4384	0.8869
**Microglia**	***C3***	**14**	**Ischemic**	1.5964	1.6697	0.0733	0.9428
**Microglia**	***Cfb***	**3**	**Control**	-3.3269	3.6562	6.9830	--
**Microglia**	***Cfb***	**3**	**Ischemic**	0.9835		-0.9835	--
**Microglia**	***Cfb***	**14**	**Control**	-1.9110	0.1170	2.0280	0.3490
**Microglia**	***Cfb***	**14**	**Ischemic**	1.0962	0.7287	-0.3675	0.7076
**Microglia**	***Cfd***	**3**	**Control**	-5.1225	-7.7605	-2.6380	0.2408
**Microglia**	***Cfd***	**3**	**Ischemic**	-7.3497	-12.2035	-4.8539	**0.0494**
**Microglia**	***Cfd***	**14**	**Control**	-7.9227	-4.9123	3.0104	0.2996
**Microglia**	***Cfd***	**14**	**Ischemic**	-6.4427	-7.7490	-1.3063	0.5903
**Microglia**	***Cfh***	**3**	**Control**	-0.8964	0.5093	1.4057	**0.0046**
**Microglia**	***Cfh***	**3**	**Ischemic**	-0.8967	-1.1682	-0.2715	0.4499
**Microglia**	***Cfh***	**14**	**Control**	0.2234	2.3168	2.0935	0.3963
**Microglia**	***Cfh***	**14**	**Ischemic**	1.1136	0.7067	-0.4069	0.2704
**Microglia**	***Cfi***	**3**	**Control**	-5.1787	-4.2258	0.9528	0.3952
**Microglia**	***Cfi***	**3**	**Ischemic**	-6.6886	-6.6432	0.0454	0.9728
**Microglia**	***Cfi***	**14**	**Control**	-5.9388	-4.2039	1.7348	0.0593
**Microglia**	***Cfi***	**14**	**Ischemic**	-5.0866	-4.1795	0.9071	0.4228
**Microglia**	***Cfp***	**3**	**Control**	-2.4964	-1.9977	0.4987	0.5124
**Microglia**	***Cfp***	**3**	**Ischemic**	1.6709	0.5607	-1.1103	0.0587
**Microglia**	***Cfp***	**14**	**Control**	-2.5868	-1.0568	1.5300	0.1463
**Microglia**	***Cfp***	**14**	**Ischemic**	0.2704	0.1984	-0.0720	0.9256
**Müller Cells**	***C3***	**3**	**Control**	-1.1192	-1.4517	-0.3325	0.7493
**Müller Cells**	***C3***	**3**	**Ischemic**	1.1076	0.9498	-0.1578	0.6577
**Müller Cells**	***C3***	**14**	**Control**	-0.5194	1.3927	1.9121	**0.0243**
**Müller Cells**	***C3***	**14**	**Ischemic**	1.8275	3.0144	1.1869	0.0589
**Müller Cells**	***Cfb***	**3**	**Control**	-2.9973	-2.8122	0.1851	0.8691
**Müller Cells**	***Cfb***	**3**	**Ischemic**	-1.0299	-0.1575	0.8725	0.0681
**Müller Cells**	***Cfb***	**14**	**Control**	-3.4479	-2.5248	0.9231	0.5226
**Müller Cells**	***Cfb***	**14**	**Ischemic**	-1.1829	-0.5403	0.6427	0.5129
**Müller Cells**	***Cfd***	**3**	**Control**	-6.5176	-9.4391	-2.9216	0.3002

**Müller Cells**	***Cfd***	**3**	**Ischemic**	-9.0726	-9.2717	-0.1991	--
**Müller Cells**	***Cfd***	**14**	**Control**	-8.0431	-9.3583	-1.3151	0.4989
**Müller Cells**	***Cfd***	**14**	**Ischemic**	-8.8784	-5.9296	2.9488	0.2882
**Müller Cells**	***Cfh***	**3**	**Control**	-1.3348	-1.0298	0.3050	0.2640
**Müller Cells**	***Cfh***	**3**	**Ischemic**	-2.5357	-1.9381	0.5976	0.2131
**Müller Cells**	***Cfh***	**14**	**Control**	-1.1971	-0.5797	0.6174	0.0742
**Müller Cells**	***Cfh***	**14**	**Ischemic**	-0.6509	-0.3095	0.3414	0.3035
**Müller Cells**	***Cfi***	**3**	**Control**	-5.6757	-7.4607	-1.7849	0.3762
**Müller Cells**	***Cfi***	**3**	**Ischemic**	-6.2857	-7.3177	-1.0320	0.1044
**Müller Cells**	***Cfi***	**14**	**Control**	-7.6991	-6.8670	0.8321	0.3035
**Müller Cells**	***Cfi***	**14**	**Ischemic**	-6.1561	-5.6158	0.5404	0.2015
**Müller Cells**	***Cfp***	**3**	**Control**	-2.9230	-3.3781	-0.4551	0.1391
**Müller Cells**	***Cfp***	**3**	**Ischemic**	-4.2322	-3.8483	0.3839	0.1619
**Müller Cells**	***Cfp***	**14**	**Control**	-3.1376	-2.7324	0.4051	0.3344
**Müller Cells**	***Cfp***	**14**	**Ischemic**	-3.4938	-3.3325	0.1613	0.7181
**Neurons**	***C3***	**3**	**Control**	-5.2992	-7.1523	-1.8532	0.1508
**Neurons**	***C3***	**3**	**Ischemic**	-1.1912	-4.0055	-2.8142	**0.0100**
**Neurons**	***C3***	**14**	**Control**	-5.9962	-5.1694	0.8268	0.5872
**Neurons**	***C3***	**14**	**Ischemic**	-5.1828	-4.0796	1.1032	0.2576
**Neurons**	***Cfb***	**3**	**Control**	-3.5318	-4.7471	-1.2153	**0.0153**
**Neurons**	***Cfb***	**3**	**Ischemic**	-2.9066	-4.5725	-1.6659	**0.0153**
**Neurons**	***Cfb***	**14**	**Control**	-4.8694	-5.4002	-0.5308	0.8284
**Neurons**	***Cfb***	**14**	**Ischemic**	-6.3055	-6.2948	0.0107	0.9961
**Neurons**	***Cfd***	**3**	**Control**	-11.3754	-12.3526	-0.9772	0.4270
**Neurons**	***Cfd***	**3**	**Ischemic**		-13.6300	-13.6300	--
**Neurons**	***Cfd***	**14**	**Control**	-10.6014	-13.9682	-3.3668	**0.0402**
**Neurons**	***Cfd***	**14**	**Ischemic**	-12.3602	-11.0411	1.3191	0.6339
**Neurons**	***Cfd***	**3**	**Control**	-4.9610	-6.2199	-1.2590	0.0615
**Neurons**	***Cfh***	**3**	**Ischemic**	-4.9317	-7.2242	-2.2925	**0.0143**
**Neurons**	***Cfh***	**14**	**Control**	-5.5778	-6.1558	-0.5781	0.1758
**Neurons**	***Cfh***	**14**	**Ischemic**	-6.8643	-5.4502	1.4141	**0.0185**
**Neurons**	***Cfh***	**3**	**Control**	-3.3386	-5.1627	-1.8241	**0.0142**
**Neurons**	***Cfi***	**3**	**Ischemic**	-3.2678	-4.6208	-1.3531	**0.0455**

**Neurons**	***Cfi***	**14**	**Control**	-6.5665	-5.7731	0.7933	0.2299
**Neurons**	***Cfi***	**14**	**Ischemic**	-5.4552	-5.8647	-0.4095	0.5497
**Neurons**	***Cfi***	**3**	**Control**	-1.9795	-2.9706	-0.9911	0.0712
**Neurons**	***Cfp***	**3**	**Ischemic**	-2.5492	-3.4375	-0.8883	0.1141
**Neurons**	***Cfp***	**14**	**Control**	-2.8764	-1.7539	1.1225	0.2957
**Neurons**	***Cfp***	**14**	**Ischemic**	-2.0179	-2.9763	-0.9584	0.5485

Table S3: Summary of statistical analysis (unpaired t-test) of the qPCR assay testing for differences of ischemic vs control samples. FC: Fold change, dct: delta CT, ddCt: delta delta CT

Fraction	Target	dpi	Treatment	Control	Ischemic	FC (ddCt)	P-Value
Microglia	*C3*	3	GFP	-4.393379	0.14019489	4.533573786	**0.0001817**
Microglia	*C3*	3	FH1-4^19-20	-2.683279	0.00635862	2.689637423	**0.0485033**
Microglia	*C3*	3	FH1-7^19-20	-2.683407	-1.0619891	1.621418238	0.1113434
Microglia	*C3*	14	GFP	1.3203053	1.59639359	0.276088238	0.9168176
Microglia	*C3*	14	FH1-4^19-20	-2.805287	-0.7536783	2.051609039	0.1877353
Microglia	*C3*	14	FH1-7^19-20	0.88188	1.6696846	0.787804556	0.5398467
Microglia	*Cfb*	3	GFP	-3.326877	0.9835345	4.310411771	**0.0155624**
Microglia	*Cfb*	3	FH1-4^19-20	-1.860932	0.63507366	2.496005297	**0.0444759**
Microglia	*Cfb*	3	FH1-7^19-20	3.6561508			--
Microglia	*Cfb*	14	GFP	-1.910987	1.09615016	3.007136742	**0.03498**
Microglia	*Cfb*	14	FH1-4^19-20	-2.947466	-1.5012318	1.446234306	0.4737238
Microglia	*Cfb*	14	FH1-7^19-20	0.1170232	0.72866201	0.611638864	0.7243838
Microglia	*Cfd*	3	GFP	-5.122509	-7.3496766	-2.227168083	**0.0336529**
Microglia	*Cfd*	3	FH1-4^19-20	-6.64631	-5.3207661	1.325543722	0.6781631
Microglia	*Cfd*	3	FH1-7^19-20	-7.760512	-12.203529	-4.443017006	0.1451675
Microglia	*Cfd*	14	GFP	-7.922664	-6.4426502	1.480014165	0.5823542
Microglia	*Cfd*	14	FH1-4^19-20	-6.88902	-5.0211989	1.867821376	0.3918623
Microglia	*Cfd*	14	FH1-7^19-20	-4.912296	-7.7489503	-2.836654027	0.2461405
Microglia	*Cfh*	3	GFP	-0.896371	-0.8966958	-0.000324885	0.9989076
Microglia	*Cfh*	3	FH1-4^19-20	-0.660342	-0.6722326	-0.01189065	0.9853815
Microglia	*Cfh*	3	FH1-7^19-20	0.5092943	-1.1681526	-1.677446842	**0.0024781**
Microglia	*Cfh*	14	GFP	0.2233527	1.11360722	0.890254545	**0.0249858**
Microglia	*Cfh*	14	FH1-4^19-20	-0.139835	0.03021932	0.170054674	0.8348011
Microglia	*Cfh*	14	FH1-7^19-20	2.3168037	0.70673676	-1.610066938	0.4529793
Microglia	*Cfi*	3	GFP	-5.178653	-6.6885796	-1.509926796	0.2113621
Microglia	*Cfi*	3	FH1-4^19-20	-5.247459	-7.3656881	-2.11822931	**0.0244022**
Microglia	*Cfi*	3	FH1-7^19-20	-4.225808	-6.6432056	-2.417397976	0.182445
Microglia	*Cfi*	14	GFP	-5.938752	-5.0866201	0.852132082	0.5898703
Microglia	*Cfi*	14	FH1-4^19-20	-4.500495	-5.0441997	-0.543704748	0.5644757
Microglia	*Cfi*	14	FH1-7^19-20	-4.20393	-4.1794834	0.024446726	0.9690711
Microglia	*Cfp*	3	GFP	-2.496416	1.67094008	4.167356491	**0.0022874**
Microglia	*Cfp*	3	FH1-4^19-20	-2.508896	0.19033241	2.699228048	**0.018183**
Microglia	*Cfp*	3	FH1-7^19-20	-1.997747	0.56067443	2.558421135	**0.0041341**
Microglia	*Cfp*	14	GFP	-2.586801	0.27035732	2.857158613	**0.0070015**
Microglia	*Cfp*	14	FH1-4^19-20	-1.754667	-0.5801342	1.174532747	0.3849348
Microglia	*Cfp*	14	FH1-7^19-20	-1.056808	0.1983736	1.255181122	0.2009603
Müller Cells	*C3*	3	GFP	-1.119152	1.10759258	2.226744652	**0.0060876**
Müller Cells	*C3*	3	FH1-4^19-20	-0.579512	1.40583134	1.985342979	0.0983368

Müller Cells	*C3*	3	FH1-7^19-20	-1.451675	0.9497664	2.401441336	**0.027245**
Müller Cells	*C3*	14	GFP	-0.519406	1.82745209	2.346857643	**0.0083029**
Müller Cells	*C3*	14	FH1-4^19-20	-1.000463	2.13285694	3.133319473	**0.00001248**
Müller Cells	*C3*	14	FH1-7^19-20	1.3926712	3.01436901	1.621697807	**0.0199047**
Müller Cells	*Cfb*	3	GFP	-2.997262	-1.0299365	1.967325052	**0.0474116**
Müller Cells	*Cfb*	3	FH1-4^19-20	-2.046833	-1.2180927	0.828740676	0.3492165
Müller Cells	*Cfb*	3	FH1-7^19-20	-2.812194	-0.1574793	2.654714346	**0.0088197**
Müller Cells	*Cfb*	14	GFP	-3.447865	-1.18293	2.264935017	**0.0353383**
Müller Cells	*Cfb*	14	FH1-4^19-20	-2.746558	-1.397836	1.34872214	0.159095
Müller Cells	*Cfb*	14	FH1-7^19-20	-2.524765	-0.5402699	1.984494686	0.2104307
Müller Cells	*Cfd*	3	GFP	-6.517577	-9.0725784	-2.555001577	--
Müller Cells	*Cfd*	3	FH1-4^19-20	-3.63897	-8.5325991	-4.893629233	**0.0220777**
Müller Cells	*Cfd*	3	FH1-7^19-20	-9.439129	-9.2716627	0.167465925	0.9539786
Müller Cells	*Cfd*	14	GFP	-8.043131	-8.8784456	-0.83531456	0.7468406
Müller Cells	*Cfd*	14	FH1-4^19-20	-7.561097	-7.3351065	0.225990423	0.9215847
Müller Cells	*Cfd*	14	FH1-7^19-20	-9.35826	-5.9296276	3.428632545	0.1304888
Müller Cells	*Cfh*	3	GFP	-1.3348	-2.5357161	-1.200915972	0.0883815
Müller Cells	*Cfh*	3	FH1-4^19-20	-1.041517	-2.0900233	-1.048505783	**0.0108474**
Müller Cells	*Cfh*	3	FH1-7^19-20	-1.029849	-1.9381187	-0.908269882	**0.001087**
Müller Cells	*Cfh*	14	GFP	-1.19711	-0.6508684	0.546241379	0.0746838
Müller Cells	*Cfh*	14	FH1-4^19-20	-0.961334	-0.6315462	0.329787731	0.4289542
Müller Cells	*Cfh*	14	FH1-7^19-20	-0.579697	-0.3094751	0.270222092	0.450025
Müller Cells	*Cfi*	3	GFP	-5.675723	-6.285676	-0.609952927	0.1444251
Müller Cells	*Cfi*	3	FH1-4^19-20	-4.866487	-5.5856843	-0.719197035	0.6058772
Müller Cells	*Cfi*	3	FH1-7^19-20	-7.460657	-7.3176799	0.142976761	0.9135902

Müller Cells	*Cfi*	14	GFP	-7.699121	-6.1561333	1.542987728	0.0599627
Müller Cells	*Cfi*	14	FH1-4^19-20	-6.311105	-4.8482602	1.462844928	0.3564768
Müller Cells	*Cfi*	14	FH1-7^19-20	-6.867013	-5.6157546	1.251257992	**0.0362905**
Müller Cells	*Cfp*	3	GFP	-2.923042	-4.2322474	-1.309205691	**0.0017758**
Müller Cells	*Cfp*	3	FH1-4^19-20	-2.795978	-3.9520264	-1.156048298	**0.0194332**
Müller Cells	*Cfp*	3	FH1-7^19-20	-3.378099	-3.8483078	-0.470208645	0.1288684
Müller Cells	*Cfp*	14	GFP	-3.137567	-3.493811	-0.356243706	0.3552183
Müller Cells	*Cfp*	14	FH1-4^19-20	-2.835665	-2.7131271	0.122537422	0.6664352
Müller Cells	*Cfp*	14	FH1-7^19-20	-2.732435	-3.3325222	-0.600087118	0.2362151
Neurons	*C3*	3	GFP	-5.299162	-1.1912236	4.107938766	**0.025373**
Neurons	*C3*	3	FH1-4^19-20	-6.170654	-3.1841843	2.986469984	**0.0220291**
Neurons	*C3*	3	FH1-7^19-20	-7.152337	-4.0054641	3.146872997	**0.0129867**
Neurons	*C3*	14	GFP	-5.996216	-5.1827965	0.813419914	0.5381086
Neurons	*C3*	14	FH1-4^19-20	-7.172916	-3.9319841	3.240932274	**0.0005179**
Neurons	*C3*	14	FH1-7^19-20	-5.169433	-4.0796206	1.089812803	0.4020806
Neurons	*Cfb*	3	GFP	-3.531822	-2.9065547	0.625267029	0.2015329
Neurons	*Cfb*	3	FH1-4^19-20	-3.699947	-3.7141485	-0.014201482	0.9859596
Neurons	*Cfb*	3	FH1-7^19-20	-4.747126	-4.5724797	0.174646616	0.6467918
Neurons	*Cfb*	14	GFP	-4.869369	-6.3055489	-1.436180353	0.6040334
Neurons	*Cfb*	14	FH1-4^19-20	-3.542999	-4.2956937	-0.752694448	0.3187766
Neurons	*Cfb*	14	FH1-7^19-20	-5.400183	-6.294823	-0.894640287	0.6585253
Neurons	*Cfd*	3	GFP	-11.37537			--
Neurons	*Cfd*	3	FH1-7^19-20	-12.35257	-13.629989	-1.277421713	0.0908159
Neurons	*Cfd*	3	FH1-4^19-20	-10.34372	-2.0669732	8.276750088	**0.0343398**
Neurons	*Cfd*	14	GFP	-10.60137	-12.360177	-1.758804957	0.5345966
Neurons	*Cfd*	14	FH1-4^19-20	-9.842963	-9.9296446	-0.086681843	0.97354
Neurons	*Cfd*	14	FH1-7^19-20	-13.96821	-11.041055	2.927159437	0.0512799
Neurons	*Cfh*	3	GFP	-4.960989	-4.9317303	0.029258966	0.9630307
Neurons	*Cfh*	3	FH1-4^19-20	-4.090824	-5.2145193	-1.123695374	0.1880955
Neurons	*Cfh*	3	FH1-7^19-20	-6.21995	-7.2242391	-1.00428915	0.1066519
Neurons	*Cfh*	14	GFP	-5.57776	-6.8642902	-1.286530113	**0.0326775**
Neurons	*Cfh*	14	FH1-4^19-20	-5.03763	-6.0344866	-0.996856117	0.204831
Neurons	*Cfh*	14	FH1-7^19-20	-6.155829	-5.4501579	0.705671358	0.0502096
Neurons	*Cfi*	3	GFP	-3.338631	-3.2677541	0.070876598	0.9390676
Neurons	*Cfi*	3	FH1-4^19-20	-4.130473	-3.1599054	0.970567465	0.1497691
Neurons	*Cfi*	3	FH1-7^19-20	-5.162738	-4.6208413	0.541896343	**0.0465596**
Neurons	*Cfi*	14	GFP	-6.566451	-5.4551613	1.111289406	0.0559243

Neurons	*Cfi*	14	FH1-4^19-20	-5.780433	-6.1630752	-0.382642317	0.7874792
Neurons	*Cfi*	14	FH1-7^19-20	-5.773131	-5.8646848	-0.091554022	0.9081472
Neurons	*Cfp*	3	GFP	-1.979481	-2.5492039	-0.569722652	0.5133704
Neurons	*Cfp*	3	FH1-4^19-20	-1.981976	-2.3773749	-0.395399332	0.3789853
Neurons	*Cfp*	3	FH1-7^19-20	-2.970581	-3.4375339	-0.466953278	**0.0035721**
Neurons	*Cfp*	14	GFP	-2.876419	-2.0179483	0.858470726	0.5637061
Neurons	*Cfp*	14	FH1-4^19-20	-2.358586	-2.6537714	-0.295185804	0.3673317
Neurons	*Cfp*	14	FH1-7^19-20	-1.753875	-2.9763188	-1.222444248	0.3176823
